# *In silico* and *in vitro* characterisation and affinity maturation of human red blood cell binding aptamers†

**DOI:** 10.1039/d5ra00645g

**Published:** 2025-07-01

**Authors:** Hayley Costanzo, James Gooch, Nunzianda Frascione

**Affiliations:** a King's College London, Department of Analytical, Environmental & Forensic Sciences London SE1 9NH UK nunzianda.frascione@kcl.ac.uk

## Abstract

Aptamers are short, single-stranded DNA or RNA oligonucleotides that can specifically bind to their target with high affinity and specificity. Aptamers have gained widespread attention in recent years as possible replacements for antibodies within many analytical fields, due to their high chemical and thermal stability and relative low cost of production. Red blood cells are of interest within not only the medical field, but also are of interest within forensic science. Few aptamers have been reported that can specifically detect human red blood cells, or surface proteins of, but they have great potential for use as biorecognition elements within immunoassays or biosensors. Three aptamers have been identified from recent literature that have been designed to bind to human red blood cells as a whole cell target or glycophorin A as a protein-based target. However, they are yet to be fully characterised for their binding affinity to red blood cells, and no sequence optimisation has been conducted. Within this work, a comprehensive characterisation of three reported aptamers has been conducted. *In silico* modelling has been explored as a means to better understand the 3D structures and the target ligand of each aptamer. The 3D structures of these aptamers have been reported and utilised within the HDOCK server to predict the docking of the aptamers to red blood cell-specific surface proteins. Both enzyme-linked oligonucleotide assays and microscale thermophoresis have been used to characterise aptamer-target biding, with dissociation constants being predicted in the nanomolar to low micromolar range for each aptamer. Additionally, sequence optimisation has been conducted to enhance the binding of the sequences to human red blood cells through sequence truncation mechanisms. To the best of our knowledge, this work represents the first characterisation of these aptamers and will guide future use of these aptamers as analytical probes.

## Abstract 2

The presented article outlines the first full characterisation and optimisation of three aptamers targeting human red blood cells, confirming their binding affinities and subsequent affinity maturation through truncation for improved analytical use.

## Graphical abstract

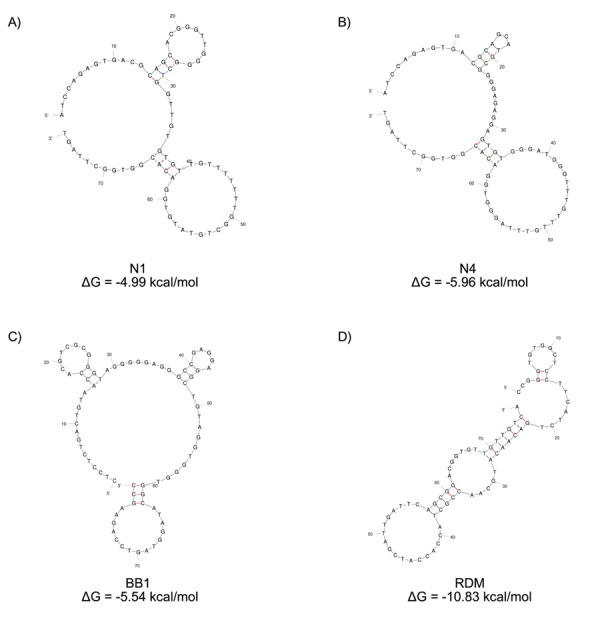

## 1 Introduction

Erythrocytes, more commonly known as red blood cells (RBCs), are a vital cell type within whole blood responsible for the transportation of oxygen and metabolic waste between the lungs and other cell types and tissues.^1^ Their structure is evolved to have a biconcave, discoid shape which permits for their flexibility when travelling throughout the body.^2^ With an average diameter of ∼7 μm, they have a large surface area and are anucleate, which contributes to sufficient gas exchange *via* the presence of haemoglobin 3. The surface proteome of a human red blood cell is highly diverse, but is dominated by a small number of abundant blood group active proteins.^4^ RBCs are of significant interest across several analytical fields and their identification and detection play a role in medical diagnostics and forensic science.^5,6^ Within forensic science, currently employed methods for the detection of human blood at crime scenes often rely on the presence of haemoglobin. Tests such as the Kastle–Meyer test rely on the peroxidase-like activity of haem to catalyse an oxidation reaction that results in an observable colour change.^7^ Despite widespread use, these tests have certain drawbacks, such as a high rate of false positives with any peroxidase containing reagents. There is a need for an analytical tool to detect human red blood cells that is able to bind to the cells with a high affinity and lack of cross-reactivity.

Aptamers are short, single-stranded DNA or RNA oligonucleotides that can specifically bind their target with high affinity and specificity.^8^ They are generated *via* the Systematic Evolution of Ligands by Exponential Enrichment (SELEX) and can be designed to bind to a wide range of targets, from small inorganic molecules to entire cells.^9^ With dissociation constants from the low micromolar to picomolar range, aptamers are comparable than some monoclonal antibodies.^9^ This high affinity is attributed to the fact that aptamers are capable of folding into stable, complex stem loop and internal loop structures upon target binding.^10^ Within analytical and diagnostic applications, aptamers are emerging as a promising alternative to antibodies due to their affinity, but also due to their modification capabilities, limited batch-to-batch variation and relative ease and lower cost of synthesis.^9^ The use of aptamers within sensors as the recognition biocomponent has been of recent interest within many medical and analytical fields.^11–17^ Whilst antibodies remain the reagent of choice in many assays due to their commercial availability and widespread use, novel aptamer research is highlighting just how significant these short strands of DNA could be if effectively harnessed.^10^

Prior to the use of an aptamer as a biorecognition element, a full characterisation of the aptamer must be conducted to ensure it is suitable for use. Whilst aptamers can have both a high affinity and specificity, the specificity of binding must be tested experimentally to ensure no cross-reactivity or non-specific binding occurs. This becomes critical when considering applications such as drug identification or analysis, where many metabolites or drugs possess extremely similar chemical structures. Therefore, having an aptamer that is able to distinguish between them is vital. This is also crucial when considering medical applications, such as cancer cell identification, where an aptamer must be able to distinguish cancerous cells from healthy ones.

Within this study, three aptamer candidates have been used that have demonstrated their ability to bind to red blood cells, as well as a randomised sequence (Table 1).^18,19^ The first two candidates, termed N1 and N4, were previously developed within the research group. They were generated *via* a modified Cell-SELEX methodology using whole human red blood cells as the target. Both N1 and N4 are 76 nucleotides in length, with the randomised region being 40 bases in length. The third candidate, referred to as BB1 within this study, was identified through a partially robotic selection using human glycophorin A (a major sialoglycoprotein on the erythrocyte membrane) as the target. BB1 is 80 nucleotides in length, with the randomised region being 40 bases in length. The randomised sequence, termed RDM, was generated through a randomised DNA sequence generator.^20^

**Table 1:** Aptamer candidates selected for characterisation and affinity maturation. Aptamer sequences are outlined by their unique identifier as well as their designed target, length in nucleotide bases and sequence

Aptamer identifier	Target	Length (bases)	Sequence (5′ – 3′)	References
N1	Whole red blood cells	76	ATCCAGAGTGACGCAGCACGGGTTGGGGCTGGTTGTGTGTTGTTTTTTTGGCTGTATGTGGACACGGTGGCTTAGT	18
N4	Whole red blood cells	76	ATCCAGAGTGACGCAGCATGCGGGGAGAGGAGTGTGGGATGGGTTTGTTTGTTTAGGGTGGACACGGTGGCTTAGT	18
BB1	Glycophorin A	80	CTCCTCTGACTGTAACCACGTCGCGGGTAGGGGGAGGGCCGAGGAGGCTGTAGGTGGGTGGCATAGGTAGTCCAGAAGCC	19
RDM	—	76	CCGGGTGTGGCTCCTTCATCTGACAACATGCAACCGCTACCACCATCGATTGATTCAGCGGACGGTGTTGTTGTCA	20

Whilst preliminary structure analysis and binding assays for N1, N4 and BB1 have been reported, a full characterisation of these aptamers, including predicted dissociation constants (*K*_D_), has yet to be documented in the existing literature. In order for such aptamers to be considered for use within analytical probes or biosensors, further characterisation is required to ensure that their binding is quantified, and subsequently enhanced. Reporting on these additional features of the aptamers will permit their use within many fields as analytical probes, as it can assist with determining practical applications and sensitivity of such assays.

Within this study, each aptamer candidate has been subjected to *in silico* modelling in order to predict its tertiary structure and subsequent 3D structure. HDOCK has then been used as a preliminary tool for the prediction of aptamer-target docking. The aptamers then underwent Enzyme-Linked Oligonucleotide Assay (ELONA) and Microscale Thermophoresis (MST) to experimentally determine their binding to both whole red blood cells and isolated proteins, and from this, dissociation constants were determined. Finally, affinity maturation for N1, N4 and BB1 was conducted to ascertain if binding to RBCs could be enhanced through sequence truncations. Therefore, the aim of this study was to fully characterise the presented aptamer candidates and assess their binding through *in silico* modelling and two *in vitro* bioassays. Additionally, affinity maturation was explored to establish whether the binding affinity observed could be enhanced through sequence modification.

## 2 Materials & methods

### 2.1. Reagents

For the isolation of red blood cells from whole human blood, a cell wash buffer was prepared (21 mM TRIS, 4.7 mM KCl, 140.5 mM NaCl, 2 mM CaCl_2_, 1.2 mM MgSO_4_, 5.5 mM glucose, 0.5% bovine serum albumin in sterile distilled water). The solution was then adjusted to pH 7.4. 3.2% sodium citrate coagulation preservative was obtained from a BD vacutainer Plus tube (Oxford, UK).

For cell counting, Countess™ Cell Counting Chamber Slides (with Trypan Blue Stain) were obtained from Thermo Fisher Scientific (CA, USA). Saline Solution 0.9% was obtained from Severn Biotech Ltd (Kidderminster, UK).

For Circular Dichroism (CD), the BB1 aptamer was synthesised with no modification by Sigma-Aldrich (Dorset, UK). CD buffer was prepared through the addition of 5 mM MgCl_2_ to PBS. This was then filtered with a 0.2 μm filter prior to use. A black 10 mm (10 mm × 4 mm) cuvette was used for all measurements. CD was performed using a ChiraScan Plus Circular Dichroism spectrometer (Applied Photophysics, Leatherhead, UK), provided by the Biomolecular Spectroscopy Centre at King's College London.

For use within ELONA, all aptamers were synthesised with 5′ biotin groups by Sigma-Aldrich (Dorset, UK). Recombinant human glycophorin A was obtained from Stratech Scientific Ltd (UK) and Recombinant Human GLUT1 was obtained from http://Antibodies.com Ltd (UK). Nunc MaxiSorp™ flat-bottom 96-well clear plates, 3,3′,5′-tetramethylbenzidine (TMB) substrate solution, 1 M sulphuric acid solution, and streptavidin-conjugated horseradish peroxidase (SA-HRP) were all obtained from Thermo Fisher Scientific (CA, USA). Carbonate/bicarbonate buffer was prepared through the addition of 0.2 M sodium carbonate solution (4 mL) to 0.2 M sodium bicarbonate solution (11.5 mL) which was then adjusted to 50 mL with DNAse-free water and adjusted to pH 9.6. Binding buffer was prepared through the addition of 0.55 mM MgCl_2_, 0.05% Tween-20 and 1% BSA to ×1 DPBS. Blocking buffer was prepared through the addition of 0.55 mM MgCl_2_ and 1% BSA to ×1 DPBS.

For MST, all aptamers were synthesised with 5′ Cyanine-5 groups by Sigma-Aldrich (Dorset, UK). Standard MO-K022 Capillaries were also obtained (NanoTemper Technologies GmbH, Germany). For the dilution of cells, MST Buffer was prepared through the addition of 0.45 g glucose, 0.5 mL of 1 M MgCl_2_ and 50 μl Tween-20 to 100 mL DPBS. For the dilution of aptamers, a 0.05% Tween-20 buffer was prepared in DPBS.

### 2.2. Red blood cell isolation & cell counting

Human blood was obtained upon informed consent from healthy donors. All samples were stored at 4 °C until cell isolation and subsequent analysis was conducted. All donors were informed and written consent was obtained. Blood samples were obtained through a finger prick and collected in an Eppendorf tube containing 3.2% sodium citrate coagulation preservative.

Red blood cells were isolated from whole blood using a cell washing protocol.^21^ Samples were first centrifuged at 500×*g* for 10 minutes before the supernatant, including the buffy coat, was removed and discarded. The RBC pellet was resuspended in 2 mL of RBC isolation buffer and centrifuged under the same conditions. The supernatant was again removed and discarded. A total of 3 washes were performed on the sample prior to the pellet being resuspended in 500 μL of saline.

To determine cell concentration of all cell types, a countess II automated cell counter (Thermo Fisher Scientific, CA, USA) was used. Cells were prepared for counting through the addition of 10 μL of cell suspension to 10 μL of trypan blue solution before being loaded onto a countess cell counting chamber slide.

All bodily fluid sample collection and use within this study was conducted in accordance with ethical clearance granted by the King's College London Biomedical Sciences, Dentistry, Medicine and Natural & Mathematical Sciences Research Ethics Subcommittee (reference HR-17/18-5057). All research was conducted in accordance with the Human Tissue Act 2004.

### 2.3. 2D structure prediction

Mfold software was used to predict the two-dimensional structures of all aptamer sequences investigated within this study (Tables 1 and S1†).^22^ All structure predictions were estimated under the following conditions: folding temperature of 25 °C, 0.137 M Na^+^ and 0.005 M Mg^2+^, which are the conditions of the original binding event during selection.

### 2.4. QGRS mapper

QGRS Mapper software was used to predict G-quadruplex-forming regions within aptamer sequences N1, N4 and BB1.^23^ Parameters were set as following: maximum length = 30 nucleotides, minimum G-group = 2 and loop size = 0–36 nucleotides.

### 2.5. 3D DNA structure predictions

The secondary structures with the lowest Gibbs free energy that were identified through Mfold software were initially converted to their respective dot bracket notations (Vienna output format). These were then used to input into RNA composer to determine the 3D RNA structure of the aptamer.^24,25^ The outputted structures were then inputted to the RNA to DNA script by Lorenz *et al.* which transforms the RNA structure to the DNA structure through energy refinement steps. The RNA to DNA conversion script can be accessed here: https://github.com/Lorenz-Lab-KCL/rna_to_dna/tree/main.^26^

### 2.6. HDOCK docking simulations

HDOCK online server was used to predict docking of N1, N4 and BB1 with prominent RBC surface proteins.^27^ Protein structures were obtained from the Protein Data Bank^28^ and aptamer structures were generated as previously outlined. Aptamer and protein structures were uploaded to HDOCK as .pdb files and default parameters were used for all simulations.

### 2.7. Circular dichroism (CD) spectroscopy

Aptamer BB1 was diluted to a stock concentration of 1 μM in CD buffer prior to being loaded to a 10 mm black cuvette (10 mm × 4 mm). CD spectra of the CD buffer was also measured and subtracted from the BB1 sample spectra as background. All measurements were obtained between 400 and 200 nm with 2 mm bandwidth at 23 °C.

### 2.8. Enzyme-linked oligonucleotide assay

All aptamer sequences (Tables 1 and S1†) were synthesised with 5′ biotin groups by Sigma-Aldrich (Dorset, UK). All aptamers were then prepared to an initial stock concentration of 5 μM in binding buffer. The aptamers were then serially diluted in binding buffer down from 5 μM to 1.5625 nM. All solutions were heated to 95 °C for 5 min and snap-cooled on ice until used.

For ELONA using RBCs, a stock solution of isolated RBCs from five healthy donors was prepared to a concentration of 1.5 × 10^6^ cells per mL in carbonate/bicarbonate buffer. A volume of 100 μL of stock RBCs was added to a Nunc Maxisorp flat-bottom 96-well clear plate and left to incubate for 1 h at 37 °C to allow the RBCs to coat the wells. For ELONA using protein targets, a stock solution of each protein was prepared to a concentration of 10 nM in carbonate/bicarbonate buffer. A volume of 100 μL of protein was added to a Nunc Maxisorp flat-bottom 96-well clear plate and left to incubate for 1 h at 37 °C to allow the protein to coat the wells. Post-incubation, the plate was drained and wells were washed (×3) with wash buffer. The wells were then blocked through the addition of 200 μL blocking buffer and left to incubate for 1 h at room temperature on a rotary shaker. After this incubation, all wells were again drained and washed (×3) with wash buffer. The serial dilution of each aptamer sequence was then added to the plate (100 μL per dilution per well) before being incubated for 1 h at room temperature on a rotary shaker. Again, all wells were drained and washed (×3) to remove unbound aptamer sequences. Per well, 100 μL of 1 μg per mL SA-HRP was added and allowed to incubate for 45 min at room temperature on a rotary shaker. A final drain and wash (×3) was carried out before the addition of 100 μL TMB substrate per well which was then incubated for 20 min at room temperature on a rotary shaker. This reaction was then stopped *via* the addition of 50 μL 1 M sulphuric acid solution. The plate's absorbance readings were then taken using an Opsys MR UV-vis microplate reader (Dynex Technologies, VA, USA) at 450 nm. GraphPad Prism (version 9.5.1, GraphPad, CA, USA) was then used to analyse the data.

### 2.9. Microscale thermophoresis

Aptamer sequences (Table 1) were synthesised by Sigma-Aldrich (Dorset, UK) labelled with 5′ cyanine-5 fluorophores. All aptamers were diluted to an initial stock concentration of 100 nM in PBS supplemented with 0.05% Tween-20. These solutions were then heated to 95 °C for 5 min and snap-cooled on ice until ready to use. RBCs were isolated from pooled whole human blood from five healthy donors and then diluted in aptamer binding buffer in an 8 step 1 : 1 serial dilution from a stock concentration of 5 × 10^8^ cells per mL. For the measurements, 5 μL of each RBC dilution was added to 5 μL of 100 nM Cy5-labelled aptamer solution, which led to a final concentration of 50 nM of the aptamer solution. The mixture was allowed to incubate for 1 hour, with each mixture being prepared in triplicate. The samples were then loaded into standard MST capillaries and loaded into a Monolith NT.115 system (NanoTemper Technologies GmbH, Germany) at a temperature of 25 °C. Instrument parameters were adjusted to 15% LED power and medium MST power. MO.Affinity software version 2.2.4 (NanoTemper Technologies GmbH, Germany) was used for the analysis of binding data obtained. Data from three independently pipetted experiments was analysed using the signal from an MST on-time of 15 s. The normalised fluorescence responses were transformed to Δ*F*_norm_ (‰) values in order to correct for baseline differences. This then allowed for the data to be plotted against cell concentration in a dose response curve. This was achieved using GraphPad Prism (version 9.5.1, GraphPad, CA, USA).

### 2.10. Affinity maturation

For each seed aptamer sequence (N1, N4 and BB1), sequence optimisation was conducted through two truncation methods; the first being primer region truncations and the second being stem loop truncations. For all aptamers, both the 5′ and 3′ primer binding regions were removed sequentially and then both removed simultaneously. Additionally, all stem loop structures within the 2D structure prediction were removed sequentially. Sequences are shown in Table S1† and structures can be seen in Section 3.3.

## 3 Results & discussion

### 3.1. *In Silico* modelling

#### 3.1.1. 2D structure predictions – seed sequences

**Fig. 1:**
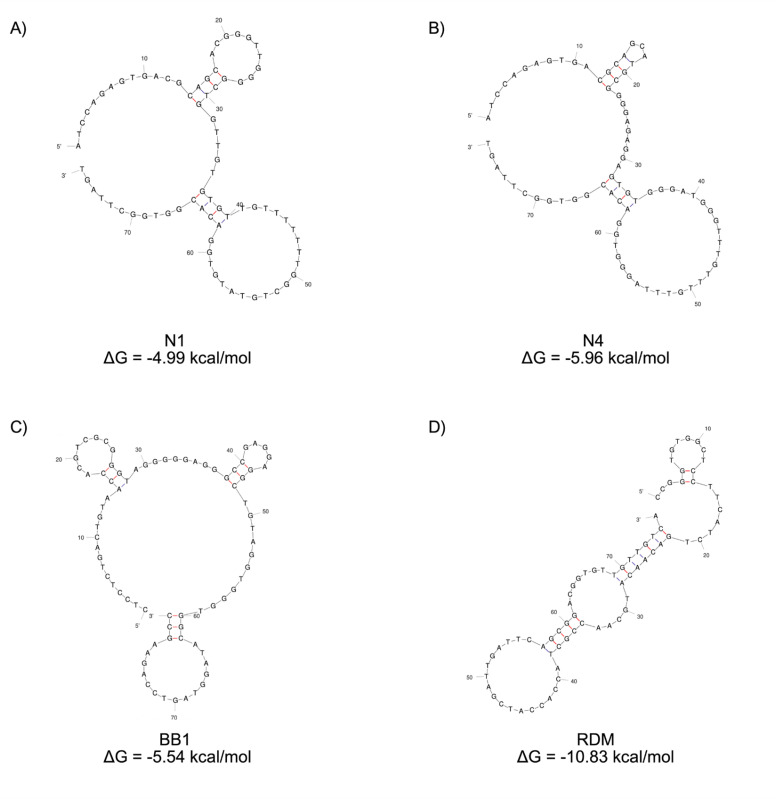
2D structure predictions of aptamers (A) N1, (B) N4, (C) BB1 and (D) RDM generated with Mfold software.^22^ The Gibbs free energy for each structure is displayed.

In order to fully characterise aptamers and perform subsequent affinity maturation, understanding the structure of the aptamers as well as their binding mechanism was vital. An aptamers structure provides stability to the complex and enhancement of binding to its target ligand through the formation of a stable aptamer–ligand complex.^29^ Therefore, Mfold – a structure prediction online software – was used to confirm the previously reported secondary structures of the aptamers included in this study under the same buffer conditions used within the binding assays (Section 2.1).^22^ These structures, as shown in Fig. 1, all display typical hairpin structures, comprising of stems and a number of loop structures. Both N1 and N4 are reported to have 2 loop structures each, with BB1 displaying 3 separate loop structures. The RDM aptamer displayed a different structure, containing 4 loop structures with two regions of internal base pairing resulting in a hairpin conformation. The probability of loop formation within each of the presented structures has also been predicted by Mfold, as shown in Fig. 1. The probability of individual nucleotides to participate in base pairs is shown through the colour of the paired bond. A red bond represents a ∼0.999 probability and a blue bond represents a ∼0.500 probability of base pairing.^22^

#### 3.1.2. 3D DNA structure predictions

**Fig. 2:**
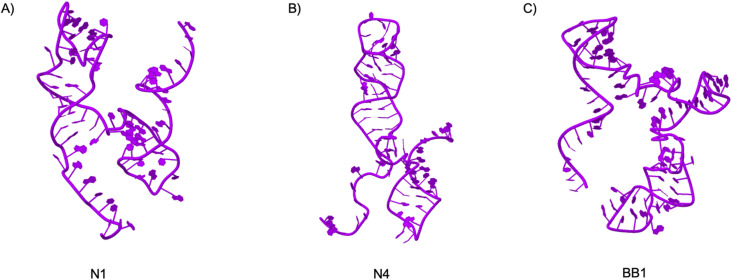
3D Structure predictions of aptamers (A) N1, (B) N4 and (C) BB1 generated using the 3D RNA to DNA pipeline reported by Lorenz *et al.*^26^ Structures visualised using Jmol.^32^

The unique 3D structure of an aptamer can be comprised of a combination of stems, hairpins, loops, pseudoknots, bulges, or G-quadruplexes.^30^ Determining the 3D structure of an aptamer can provide a molecular level understanding of the folding and dynamics of such structures.^31^ When considered the application of these aptamers, in fields such as biosensing for cell detection or imaging, gaining additional information on an aptamer's structure can guide future research into aptamer maturation and interface interactions, such as target ligand docking. This can aid in the enhancement of specificity, selectivity and stability of aptamer-target complexes.^31^ Docking simulations are a valuable tool when studying aptamer interactions as they can predict the probability of binding interactions to a range of targets. To utilise such molecular dynamic simulation software, an aptamer's 3D structure must first be predicted for the input of the molecular docking software. Despite the proven need for an automated 3D DNA modelling software, there is a limited number of platforms that are accessible for the simple conversion of a 2D to 3D structure without the need for extensive computer modelling knowledge. Therefore, the pipeline for 3D structure predictions presented within this work is a combination of existing methods for 3D RNA structure predictions using RNA Composer followed by the use of a novel pipeline RNA to DNA aptamer 3D structure conversions developed by Lorenz *et al.*^26^ For each aptamer (N1, N4 and BB1), the 3D DNA structure has been predicted (Fig. 2). These structures were therefore predicted for the purpose of subsequent docking simulations to provide insight into the possible docking of N1, N4 and BB1 with prominent RBC surface proteins.

Briefly, the secondary structures with the lowest Gibbs free energy that were identified through Mfold software were initially converted to their respective dot bracket notations (Vienna output format). These were then used to input into RNA composer to determine the 3D RNA structure of the aptamer.^24,25^ The outputted structures were then inputted to the RNA to DNA script by Lorenz *et al.*^26^

#### 3.1.3. QGRS mapper

**Fig. 3:**
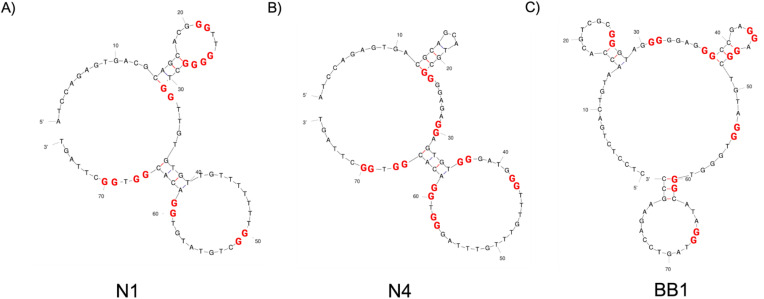
2D structure predictions of aptamers (A) N1, (B) N4 and (C) BB1 generated with Mfold software.^22^ The G-quadruplex forming regions as predicted by QGRS Mapper are highlighted through red bases.

G-quadruplexes are a class of structures that are formed by G-rich DNA oligonucleotides.^33^ With varying size and structure, they can be formed by a number of strands of DNA (one, two or four strands), with the direction also being variable (parallel or antiparallel). G-quadruplexes continue to gain interest within aptamer characterisation as they can give an indication of greater thermal and chemical stability *vs.* unstructured sequences.^18^ QGRS Mapper is an online tool used to identify G-quadruplex-forming regions within N1, N4 and BB1.^23^Table 2 and Fig. 3 outlines the position on the sequence, the length of the predicted G-quadruplex, sequence and G-score that has been determined by QGRS Mapper. The G-score is an assigned value to each quadruplex-forming guanine-rich sequence that indicates the likelihood of formation of a stable G-quadruplex.^23^ This score is calculated based on factors such as the number of G-tetrads that can form, the length and composition of loop regions between guanine tracks and the overall number of guanine tract. A higher G-score generally indicates that the sequence will form a stable G-quadruplex.

**Table 2:** QGRS Mapper predictions for N1, N4 and BB1. The position on the sequence and length of QGRS is given, as well as the sequence itself. The guanine groups that form the tetrad are in bold and underlined. The G-score given is an indication of the likelihood of a GQRS to form a stable G-quadruplex.^23^

	Position	Length	QGRS	G-score
N1	21	12	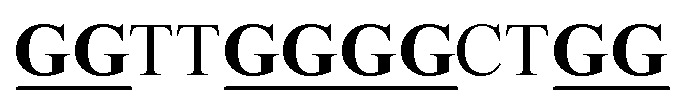	19
	50	21	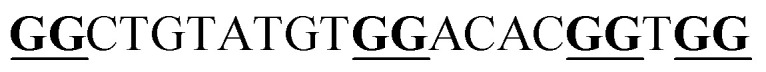	14
N4	22	22	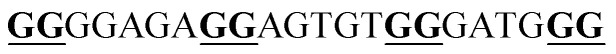	20
	57	14	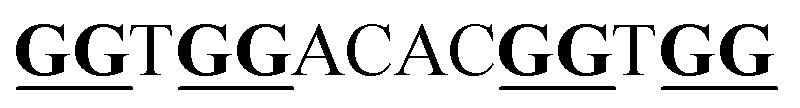	18
BB1	25	20	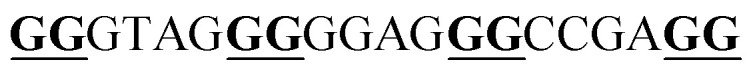	21
	46	22	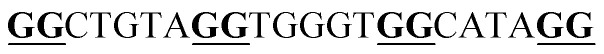	20

As previously reported during the initial selection of N1 and N4, both of these aptamers show two G-quadruplex-forming regions each. Similarly, BB1 is reported to have 2 G-quadruplex-forming regions, which have also been identified and assigned a G-score in Table 2. Reporting these regions can help to better understand the aptamer-target interaction and subsequent optimisation of the sequence.^18^

#### 3.1.4. Docking simulations

**Fig. 4:**
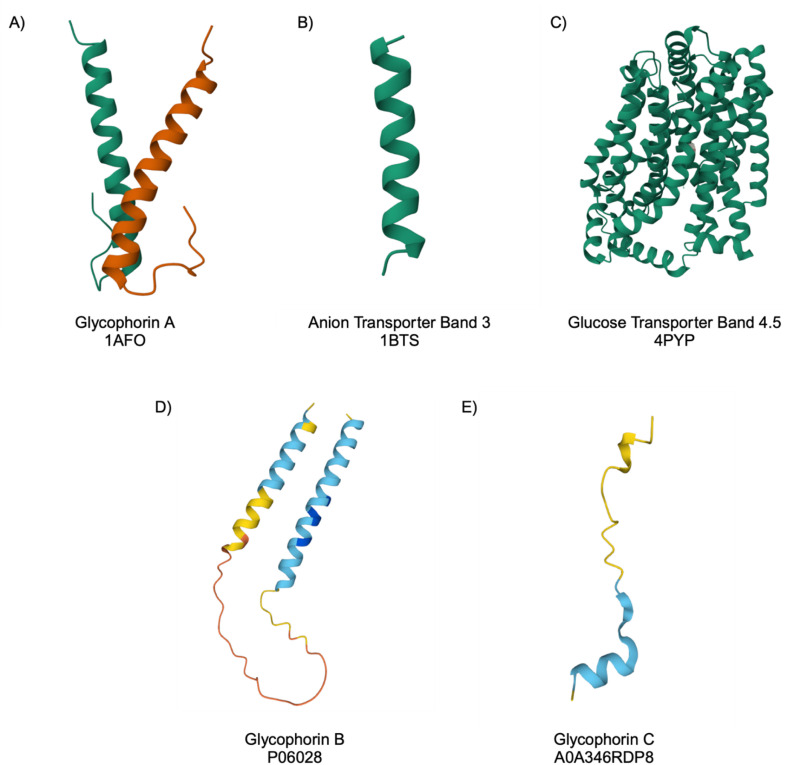
Protein structures used for docking simulations in HDOCK with aptamers N1, N4 and BB1. Protein structures shown: (A) Glycophorin A, (B) Anion Transporter Band 3, (C) Glucose Transporter Band 4.5, (D) Glycophorin B and (E) Glycophorin C. Protein structures were obtained from the Protein Data Bank^35,36,38^ and AlphaFold Protein Structure Database.^41–43^

Docking simulations can be a valuable tool when considering aptamers applications. They allow for the rapid screening of possible target binding sites, as well as to estimate any possible cross-reactivity or non-specific binding. They can save time and costs associated with experimental testing, instead helping to guide future experiment work on affinity maturation or aptamer modification. HDOCK is an online molecular dynamic docking algorithm that is able to predict the docking of protein–ligand interactions and offers a hybrid docking algorithm of template-based modelling and free docking.^27,30^ For each aptamer–protein combination trialled, a docking score and confidence level have been generated. The tertiary structures modelled for N1, N4 and BB1, HDOCK were used to screen prominent erythrocyte surface proteins for their docking ability. Proteins were selected for screening based on their copy number on the surface of the red blood cell, with 5 proteins initially selected with the greatest copy number (Table 3 and Fig. 4).

**Table 3:** Erythrocyte surface proteins selected for screening with aptamer candidates N1, N4, BB1 and RDM. An outline of the function of the protein as well as the copy number per red blood cell is given

Protein	Function	Copies/Cell	References
Glycophorin A	A major sialoglycoprotein that bears the MNSs antigens	∼1 × 10^6^	4 and 35
Anion transporter band 3	Responsible for conducting chloride/bicarbonate anion exchange across the plasma membrane	∼1 × 10^6^	4, 36 and 37
Glucose transporter band 4.5	Mediates the basal-level glucose uptakes by erythrocytes through facilitative diffusion	∼5 × 10^5^	4 and 38
Glycophorin B	A component of the ankyrin-1 complex, involved in the stability and shape of the erythrocyte membrane	2.5 × 10^5^	4 and 39
Glycophorin C	An integral membrane sialoglycoprotein that regulates the mechanical stability of erythrocytes	0.6–1.2 × 10^5^	4 and 40

The first protein chosen for HDOCK screening, and the most prolific surface protein on the red blood cell, is glycophorin A, a glycoprotein with a molecular mass reported to be between 29 and 36 kDa.^34^ The second protein selected, which is reported to be the second most abundant protein on the surface, is anion transporter band 3 with a molecular mass of 100 kDa.^34^ The third protein chosen for initial HDOCK screening was glucose transporter band 4.5, which is ubiquitously expressed on the erythrocyte surface and has a molecular mass of 55 kDa.^34^ In addition to these highly expressed proteins, two additional glycophorins were included in HDOCK screening: glycophorin B & C. Despite being similar to glycophorin A in terms of their sialic acid residues and function in blood grouping, these proteins vary in both their primary sequence and their size. Glycophorin B is 32 kDa and glycophorin C is 35 kDa, making them similar in molecular mass to the most highly expressed erythrocyte protein. Table 3 outlines their preliminary function and estimated copy number.

Given that the exact binding location of N1 and N4 remains unreported, HDOCK was deemed an appropriate tool to estimate probable binding site(s) on the red blood cell surface. For the BB1 aptamer, the target is reported as glycophorin A, so HDOCK was used to confirm this binding and assign a docking score to it. It is important to note that the HDOCK screening is not intended as a definite docking analysis, but rather a mechanism to screen possible binding sites. Whilst three prolific surface proteins have been selected for this screening, the actual target binding sites, or any additional binding sites, for N1 and N4 may fall outside of this analysis. In addition, the surface of a red blood cell is highly diverse, therefore it is possible that an aptamer may bind to multiple surface proteins. Therefore, the most abundant surface proteins were chosen for initial screening in HDOCK to give an indication as to the more probable binding sites for N1, N4 and BB1.

The docking score and confidence levels predicted by HDOCK can be seen in Table 4. The docking score given indicates the strength of docking, with a more negative score indicating a more possible binding event. Many protein–protein/RNA/DNA complexes within the Protein Data Bank generally display a docking score of −200, indicating that docking is probable.

**Table 4:** HDOCK docking estimation scores for aptamers N1, N4 and BB1 with a selection of abundant erythrocyte surface proteins. For each docking event, the docking score and confidence score has been assigned. Only the best scoring model for each aptamer–protein docking simulation has been reported^27^

	Protein	Docking score	Confidence score
N1	Glycophorin A	−237.69	0.8524
	Anion transporter band 3	−191.64	0.6969
	Glucose transporter band 4.5	−295.18	0.9480
	Glycophorin B	−239.83	0.8577
	Glycophorin C	−199.99	0.7310
N4	Glycophorin A	−237.93	0.8530
	Anion transporter band 3	−182.09	0.6552
	Glucose transporter band 4.5	−339.36	0.9778
	Glycophorin B	−220.29	0.8031
	Glycophorin C	−195.03	0.7111
BB1	Glycophorin A	−231.07	0.8350
	Anion transporter band 3	−166.18	0.5802
	Glucose transporter band 4.5	−310.48	0.9612
	Glycophorin B	−225.26	0.8183
	Glycophorin C	−184.07	0.6641

The software empirically calculates the confidence score assigned to each docking model. If the confidence score is > 0.7, the two molecules would be very likely to bind; if it is between 0.5 and 0.7 then the molecules would be possible to bind; when the confidence score is < 0.5, the two molecules are unlikely to bind.^27^ For each aptamer–protein docking simulation, only the top scoring model has been chosen for inclusion. Each aptamer–protein pair generates a possible 4392 models, however, given that HDOCK was only used for a screening rather than absolute binding production, only the greatest scoring model has been considered. Alongside the docking score assigned in Table 4, a visual representation of the docking site can be seen in Fig. S1–S3.†

The N1 aptamer, which has an unknown exact binding site on the red blood cell surface, showed the greatest docking score to glucose transporter band 4.5, with a score of −295.18 and a confidence score of 0.9480. Given the high confidence score, it can be assumed that this is a highly probably binding site for the N1 aptamer. Similarly, N1 demonstrated a likely docking with glycophorin A with a score of −237.69 and a confidence score of 0.8525, this was also observed for docking with glycophorin B, which produced a very similar docking score of −239.83 and a confidence score of 0.8577. Given the structural similarities between glycophorin A and B, it was not unexpected that similar docking scores were obtained. However, docking with glycophorin C was deemed improbable, with a predicted score of −199.99, which is not considered probable by the Protein Data Bank. Whilst the HDOCK data suggested that the docking event to glucose transporter band 4.5 would be a more likely binding event, it is highly feasible that the N1 could bind to either, or all, probable binding sites. This is likely because during the initial selection of the aptamer (*via* SELEX), the entire red blood cell was used, and given that both of these proteins display a high abundance on the surface, all could bind with the aptamer. When comparing these binding events with anion transporter band 3, it was shown that docking between N1 and this protein would be possible, but far less likely with a docking score of −191.64 and a confidence score of 0.6969.

Similarly to the N1 aptamer, the N4 aptamer demonstrated the most probable docking to glucose transporter band 4.5, producing a docking score of −339.36 and a confidence score of 0.9778. When modelled with glycophorin A and B, very likely docking events were also predicted, but with a decreased docking score of −237.93 and a confidence score of 0.8530, and −220.29 and 0.8031, respectively. Given the suggestion of a two-site binding model for the N4 aptamer also, multiple binding sites should be considered for this aptamer. Docking to the anion transporter band 3 would also be less likely to occur, as a docking score of −182.09 was predicted with a confidence score of 0.6552. This was also observed for docking with glycophorin C, where a less probable docking event was observed, with a docking score of −195.03 and a confidence score of 0.7111. The docking prediction similarities between N1 and N4 are expected, given the similarity in secondary structure between the two sequences. This, therefore, results in similar docking trends and targets between the two aptamers.

The BB1 aptamer was selected to bind to glycophorin A and has been reported to show preliminary binding data to the protein 19. Indeed, this binding was confirmed using HDOCK, as a docking score of −231.07 was obtained with a confidence score of 0.8350, validating that binding would be highly likely. However, this aptamer also demonstrated a likely docking to glucose transporter 4.5 with a docking score greater than that of glycophorin A. The docking score was −310.48 with a confidence score of 0.9612, indicating the binding is very likely. Interestingly, docking with glycophorin B resulted in a docking score of −225.26 with a confidence score of 0.8183, which suggested a very likely docking of BB1 to this protein as well. Given the similarities in sialoglycoprotein structure, this is not an unexpected docking result. Similarly to both N1 and N4 aptamers, BB1 resulted in a marginally possible binding event with anion transporter band 3, with a docking score of only −166.16 and a confidence score of 0.5802. Additionally, docking simulations with glycophorin C also suggested that binding would be far less likely, with the lowest docking score of −184.07 and confidence score of 0.6641. This indicates that the most likely docking would be to glucose transporter band 4.5 or glycophorin A.

### 3.2. *In vitro* aptamer characterisation

#### 3.2.1. Circular dichroism (CD)

**Fig. 5:**
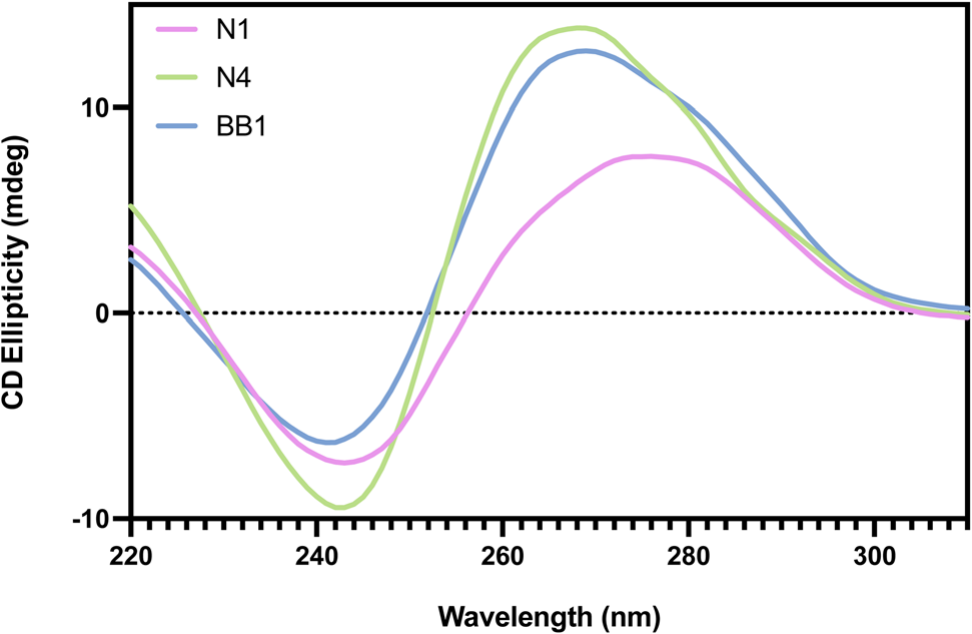
Circular dichroism spectra of aptamers N1, N4 and BB1 in their folded, unbound state.

Circular dichroism (CD) spectroscopy is a powerful biophysical technique used to study the secondary structures of chiral molecules, including aptamers.^44^ In the context of DNA aptamers, CD provides critical information about the conformational features of oligonucleotides in solution, such as the formation of G-quadruplexes, hairpins, or duplexes. As aptamer function is highly dependent on the adoption of specific three-dimensional conformations, determining the presence and stability of such structures under experimental conditions is vital. By providing rapid, label-free insight into structural motifs, CD complements other characterisation techniques and helps ensure that an aptamer's binding properties are directly linked to its predicted or selected structure. As shown in Fig. 5, the CD spectra shown illustrate the secondary structure characteristics of aptamers N1, N4 and BB1 in solution. Whilst N1 and N4 have previously reported spectra, the BB1 spectra was obtained under identical experimental conditions to allow for a direct comparison of spectra.^18^

For the N1 aptamer, the spectrum showed an ellipticity minima at ∼240 nm and a maxima at ∼280 nm. This spectrum is indicative of a B-DNA helix structure.^45^ The N4 and BB1 aptamers, however, both demonstrated an ellipticity minima at ∼240 nm and a maxima at ∼265 nm, which is indicative of parallel quadruplex structures.^46^ This suggests a predominance of guanine-rich quadruplex formation in both of these sequences, which is consistent with *in silico* findings. Interestingly, N4 and BB1 exhibit more intense signals than N1, suggesting a higher degree of structural ordering or greater G-quadruplex content. The subtle spectral differences between the aptamers may reflect variations in the stability or compactness of the folded structures, which can influence their binding performance and specificity. Overall, the CD data confirm that each aptamer adopts a structured conformation compatible with target recognition and provide preliminary insight into potential structural differences that could impact functional behaviour.

#### 3.2.2. Binding affinity studies

##### 3.2.2.1 Enzyme-linked oligonucleotide assay (ELONA)

**Fig. 6:**
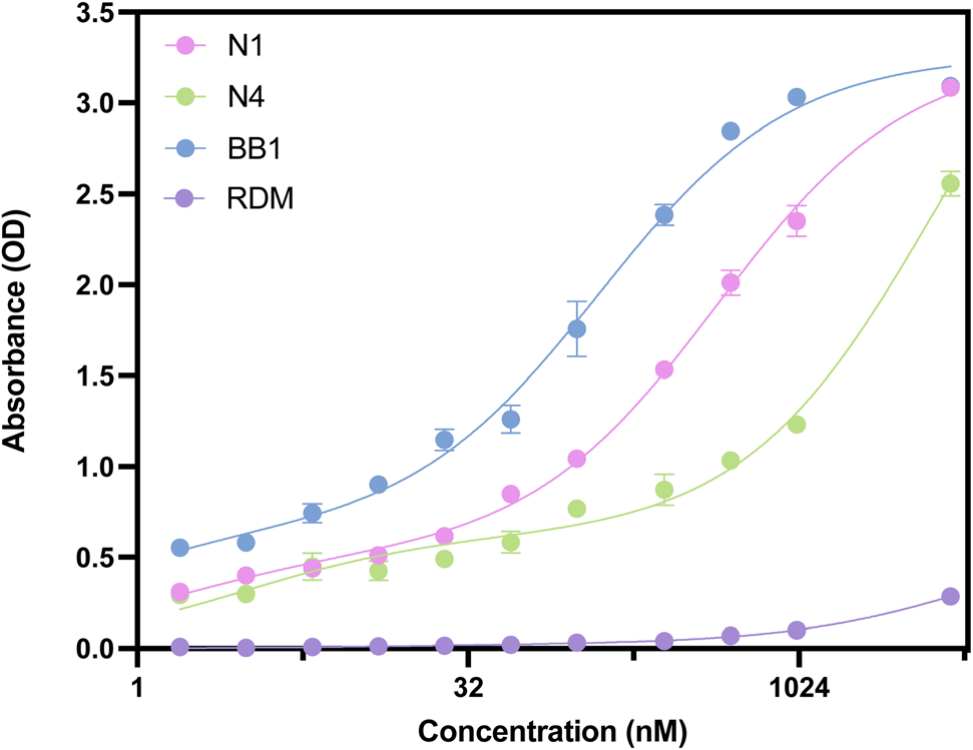
ELONA absorbance responses of interactions between a varying concentration range of aptamers N1, N4, BB1 and RDM and red blood cells. Error bars = s.d.; *n* = 4 (independent experiments).

Enzyme-linked oligonucleotide assay (ELONA) was initially used to confirm binding of each aptamer to human red blood cells and to calculate the *K*_D_. ELONA is a well-established technique for assessing aptamer-target interactions, offering a straightforward means of quantifying binding *via* a colourimetric readout. Its plate-based format allows for parallel screening of multiple aptamers and concentrations, making it well-suited for initial binding validation and affinity calculations. Although it involves immobilisation of the target, ELONA remains a widely used and reliable method within the aptamer field, particularly for verifying binding activity against whole cells in a high-throughput and comparative manner. Therefore, it was used to assess the binding of N1, N4, BB1 and RDM to RBCs. To account for intra-donor variation in expression of surface proteins, a total of five donors were used for ELONA, with whole blood being pooled prior to isolation of RBCs to diversify the pool. Isolated RBCs were initially diluted to a concentration of 1.5 × 10^6^ cells per mL, with 100 μL of solution adsorbed per well (∼150 000 cells per well). Aptamer dilutions from 5 μM–1.5625 nM were used to cover a large concentration range which therefore ensured that a binding event could be recorded for each aptamer. Whilst the concentration range used within this study is broad, this was intentional as the number of possible binding sites was unknown, so sufficient aptamer needed to be added to attempt to saturate the binding sites. In addition to the three aptamer candidates, a scrambled sequence (76 nucleotides) was also incubated with the RBC solution. This was conducted to assess if non-specific binding is observed through an unselected aptamer sequence to red blood cells. Fig. 6 shows the ELONA absorbance values obtained from the incubation of N1, N4, BB1 and the scrambled sequence (RDM) with RBCs. All three aptamer sequences demonstrate dose-dependent binding responses as expected, with an increase in aptamer concentration producing an elevated absorbance reading. The randomised sequence, RDM, did not show dose dependent binding to RBCs over the concentration range experimentally trialled.

Non-linear regression was conducted using GraphPad Prism (version 9.5.1, GraphPad, CA, USA) using a two-site specific binding model. This binding model was selected for this data as the curves obtained suggest two-site binding events are occurring during the binding of the aptamers to targets. Whilst most commonly seen within protein and enzyme binding models, a multisite binding model is less frequently observed for nucleic acid interactions. The two-site binding model suggests that an aptamer is capable of interacting with its target molecule at two distinct binding sites. These sites may differ in their affinity for the aptamer as well as their spatial arrangement on the target. A two-site binding can occur in an independent or cooperative manner, with the dissociation constants varying accordingly. Multisite target binding has been observed within notable reported aptamers, such as the cocaine-binding aptamer which binds to two independent sites on a cocaine molecule with differing affinities^47^ and an ATP binding aptamer which is able to bind two ATP molecules at separate sites.^48^ When considering the surface diversity of the target, N1 and N4 aptamers may likely bind to one or possibly more binding sites, with different affinities to each. Similarly, BB1 was reported to bind to glycophorin A and the data also suggests a two-site specific binding model. Both *K*_D_ Hi and *K*_D_ Lo values have been determined and reported in Table 5. As seen in Fig. 6, all the aptamer candidates produce a dose-dependent substrate response, with BB1 producing a greater substrate response than both N1 and N4. This is also reflected in the calculated *K*_D_ values, which show that BB1 has a lower *K*_D_ value, while N1 and N4 have higher *K*_D_ values (Table 5). It should, however, be noted that although a two-site specific binding model has been chosen for N1 and BB1, the *K*_D_ Hi value falls outside of the experimental range and, thus, cannot be reliably reported. Within this assay, extending the concentration range lower than that already investigated was not feasible due to sensitivity limitations, so the *K*_D_ Hi values could not be experimentally trialled. Therefore, the *K*_D_ Lo values have been assumed to be the most accurate calculation of the dissociation constants for these aptamers. From the values that have been reported, it has been determined that all RBC binding aptamers have a *K*_D_ value within the nanomolar to low micromolar range (*R*^2^ = 0.99), with the scrambled sequence displaying a much greater *K*_D_ value, indicating its inability to specifically bind to RBCs. Calculated aptamer *K*_D_ values within the nanomolar range are desirable and comparable, if not marginally improved, when compared with antibodies that can specifically bind cells.^49^

**Table 5:** Dissociation constants estimated for N1, N4 and BB1 when incubated with either human RBCs, glycophorin A or glucose transporter band 4.5

Target	Aptamer	*K*_D_ Hi (nM)	*K*_D_ Lo (μM)	*R* ^2^
Red blood cells	N1	1.388 ± 0.600	0.437 ± 0.037	0.998
	N4	2.958 ± 1.107	4.235 ± 1.452	0.992
	BB1	0.450 ± 0.500	0.127 ± 0.020	0.996

Furthermore, it should be noted that the aptamers used in this study, N1, N4, and BB1, have previously been demonstrated to bind their target effectively in the presence of whole blood.^18,19^ These earlier findings provide evidence that the aptamers retain specificity and functionality in complex biological environments, supporting their suitability for use in physiologically relevant conditions. As such, while this study focused on characterising the binding of these sequences with intact red blood cells under defined buffer conditions, their performance in whole blood has already been established in prior work. This further underscores their potential applicability in clinical or diagnostic settings where complex sample matrices are encountered.

The docking simulations reported within this study suggested that N1, N4 and BB1 may bind with the highest probability to glycophorin A and glucose transporter band 4.5 (Table 4). In order to experimentally evaluate these findings, ELONA was employed to assess the binding between the aptamers and these proteins and to calculate the *K*_D_. Both glycophorin A and glucose transporter band 4.5 were initially diluted to a concentration of 10 nM before being added to the plate. Aptamer dilutions were kept consistent with those reported previously for incubation with RBCs. Non-linear regression was conducted using GraphPad Prism (version 9.5.1, GraphPad, CA, USA) using a one-site specific binding model. This difference in binding model between the whole cell target and protein target could provide further insight into the binding of the aptamer to the target. The two-site binding model used for RBC incubation suggested that aptamers may likely bind to one or more binding sites on the target, which is probable given the diversity of the RBC surface. However, when incubated with the isolated protein alone, this model suggests that the aptamer is binding to one site only. This, therefore, could indicate that each aptamer is binding to multiple target proteins on the RBC surface. Multisite binding could be experimentally determined using techniques such as mutagenesis mapping, whereby site-directed mutagenesis of a protein target is conducted. Aptamer binding to the protein could then be monitoring after different site mutagenesis to ascertain if binding is inhibited.^50^ Therefore, this could allow for the protein-specific binding site to be experimentally confirmed.

**Fig. 7:**
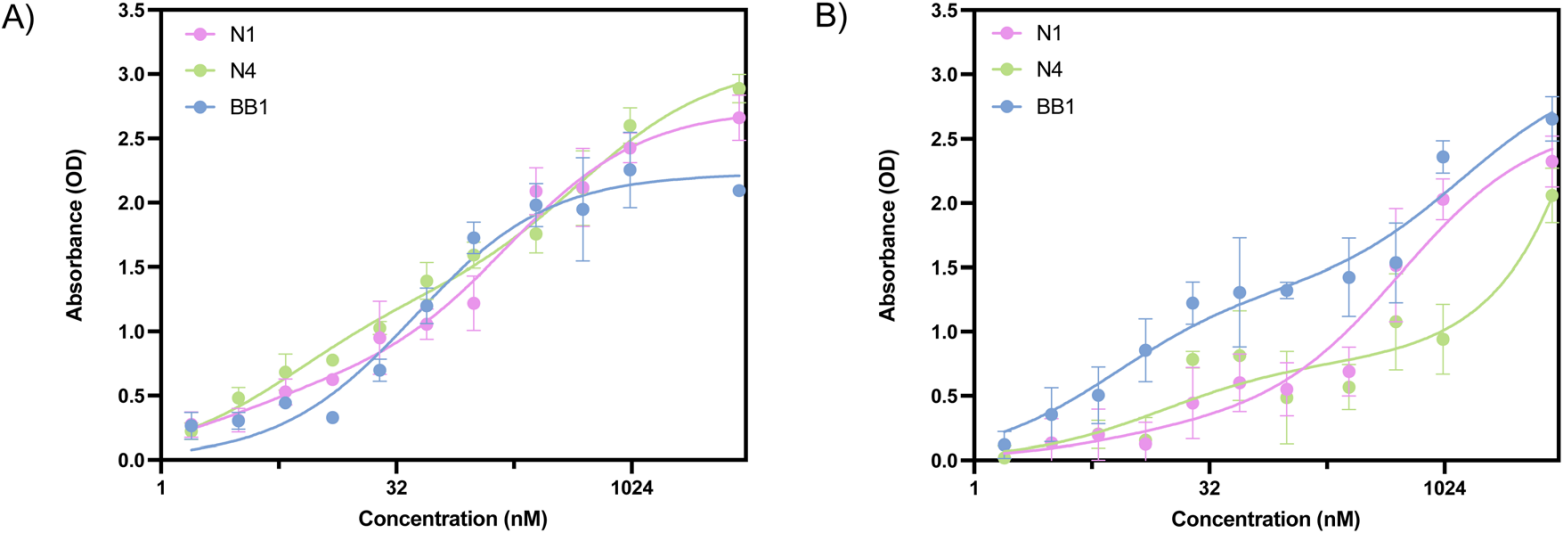
ELONA absorbance responses of interactions between a varying concentration range of aptamers N1, N4 and BB1 (A) glycophorin A and (B) glucose transporter band 4.5. Error bars = s.d.; *n* = 3 (independent experiments).

ELONA absorbance values obtained from the incubation of N1, N4 and BB1 with glycophorin A are shown in Fig. 7A. All three aptamer sequences demonstrate dose-dependent binding responses. When considering the HDOCK docking scores obtained with glycophorin A, a binding event was expected for all three aptamers. This was confirmed through ELONA, where N1, N4 and BB1 demonstrated dose-dependent binding when incubated with 10 nM glycophorin A. As previously mentioned, the BB1 aptamer was initially selected with glycophorin A as the intended target ligand. This was confirmed through ELONA, where the *K*_D_ value was calculated as 25.02 ± 10.32 nM (Table 6). This suggested that of the three aptamers, BB1 had the highest affinity to the protein. Calculated *K*_D_ values for N1 and N4 were within the low micromolar range, indicating that whilst binding occurred, the affinity of BB1 to the protein was greater than that of N1 or N4 (Table 6). Fig. 7B shows the ELONA absorbance values obtained from the incubation of N1, N4 and BB1 with glucose transporter band 4.5. Similarly to results obtained from incubation with RBCs and glycophorin A, all three aptamer sequences demonstrate dose-dependent binding responses. HDOCK docking scores indicated that of all surface proteins screened within this study, glucose transporter band 4.5 produced the greatest docking probability scores, suggesting that all three aptamers would bind to the protein. This was confirmed through ELONA, where all three aptamers resulted in a calculated *K*_D_ value within the nanomolar range (Table 6).

**Table 6:** Dissociation constants estimated for N1, N4 and BB1 when incubated with either glycophorin A or glucose transporter band 4.5

Target	Aptamer	*K*_D_ (nM)	*R* ^2^
Glycophorin A	N1	355.8 ± 95.56	0.948
	N4	354.5 ± 227.0	0.716
	BB1	25.02 ± 10.32	0.857
Glucose transporter band 4.5	N1	59.53 ± 13.62	0.954
	N4	37.57 ± 10.65	0.926
	BB1	41.60 ± 7.122	0.975

##### 3.2.2.2 Microscale thermophoresis (MST)

**Fig. 8:**
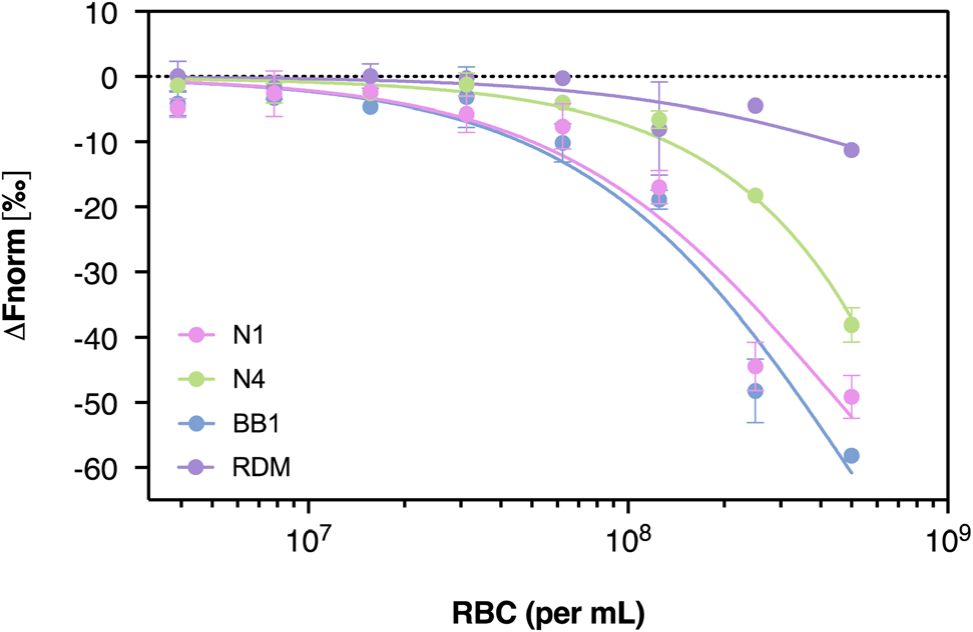
Microscale thermophoresis dose–response curves for aptamers N1, N4, BB1 and RDM against serially diluted red blood cell suspensions. Error bars = s.d.; *n* = 3 (independent experiments).

Microscale thermophoresis (MST) is an emerging technique used to study molecular interactions with high sensitivity and minimal sample consumption. When considering its use with aptamers, MST offers the key advantage of being a solution-based method, thereby eliminating the need for aptamer immobilisation, which can potentially alter structure and binding properties. This is particularly important when working with structured nucleic acids like aptamers, whose conformation is critical to target recognition. Additionally, MST is well-suited for use with complex and heterogeneous targets such as whole red blood cells, as it allows interactions to be studied under near-physiological conditions without requiring target purification or surface coupling. These features made MST an appropriate and minimally disruptive method for characterising aptamer-RBC interactions within this study. Therefore, MST was carried out to demonstrate that the binding of N1, N4, and BB1, to red blood cells is observed when using an assay whereby the red blood cells or aptamers are not immobilised. In addition to these aptamer, the randomised sequence, RDM, was also incubated with red blood cells to assess the binding of an unselected sequence against the target. Having a non-immobilised target is in contrast to the previously discussed ELONA techniques, whereby the red blood cells are immobilised to the wells of a plate, possibly altering binding events. In these MST experiments, the concentration of Cy5-labeled aptamers (N1, N4, BB1 and RDM) was kept constant (50 nM), whilst the concentration of the non-labelled RBCs was varied between 3.9 × 10^6^–5 × 10^8^ cells per mL. After a 1-hour incubation, the samples were loaded into standard Monolith NT.115 Capillaries (NanoTemper Technologies) and the MST measurement was performed using the Monolith NT.115 (NanoTemper Technologies). An MST-on time of 15 s was used for analysis demonstrating a binding event. Fig. 8 shows the baseline corrected normalised fluorescence changes (Δ*F*_norm_) for aptamers N1, N4 and BB1 as a function of red blood cell concentration.

A change in emission signal can be observed for all three aptamer sequences (N1, N4 and BB1) in response to an increase in RBC concentration. This is indicative of a binding event occurring between the target cells and each aptamer, with BB1 demonstrating the greatest response amplitude, hence the greater Δ*F*_norm_. This is consistent with the binding data obtained from ELONA (Fig. 6), with BB1 estimated to show the greatest level of binding to RBCs when compared to N1 and N4, which show a slightly decreased response. Similarly to the results obtained from ELONA, the randomised sequence (RDM) shows a lower Δ*F*_norm_, indicating a limited binding interaction between this unselected sequence and the target RBCs.

Unlike traditional use of MST, the use of whole cells as a target ligand (opposed to more commonly used proteins) means that an accurate *K*_D_ value cannot be generated. This is because the molar concentration of cells cannot be determined without using radiolabelled aptamers.^51^ One possible way to overcome this would be to use a fluorescently labelled constant concentration of cells with a concentration range of aptamer instead of using a serial dilution of RBCs and a fixed concentration of fluorescently labelled aptamer. However, finding a fluorescent stain that is stable within the RBC over a long period of time (*i.e.*, 1–2 hours) proved challenging. Given that most commercially available cell dyes bind to surface proteins or require a nucleus to achieve staining, this approach was not deemed viable as attaching fluorophores to the surface of the RBCs may interfere with aptamer binding and affect experimental reading. Also ensuring that labelling between cells is consistent would be difficult to achieve as binding sites for the dye may vary between cells. However, with the novel use of MST for whole cell-aptamer analysis, binding of the aptamer to RBCs could be confirmed for N1, N4 and BB1 (Fig. 8).

### 3.3. Affinity maturation

High binding affinity is a vital requirement of all aptamers.^52^ Despite the SELEX selection processes reported to develop N1, N4 and BB1 having stringency measures in place to ensure that the selection results in high affinity candidates, this does not necessarily mean that selected aptamers cannot be further modified to increase the strength of binding. It is reported that only part of an aptamer's sequence is critical for its function and target binding, so truncation may lead to a structure that can fold more efficiently to interact with its target.^53^ Therefore, redundant bases or regions may introduce instability in the tertiary structure and can lead to suboptimal affinity for the target in different solutions.^54^ By reducing the length of an aptamer, not only can the cost of production be reduced, but the resulting aptamer could have an increased affinity for the target and an increased performance as an analytical reagent.^55^

**Fig. 9:**
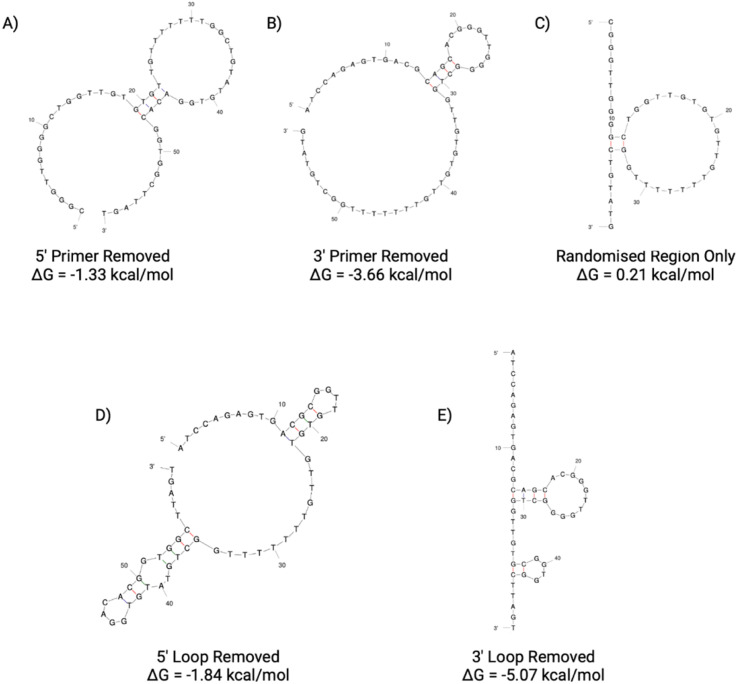
N1 aptamer truncations with the following sequence modifications: (A) 5′ primer removed, (B) 3′ primer removed, (C) 5′ and 3′ primer removed, (D) 5′ loop removed and (E) 3′ loop removed. Structure generated from Mfold software.^22^

In order to enhance the binding affinity of each aptamer to RBCs, a number of aptamer truncations were conducted. Sequence truncation can help to identify possible binding sites based on truncations made to the predicted secondary structures.^52^ To assess the impact of sequence truncation on an aptamers structure, the predicted secondary structures and corresponding Gibbs free energy values were included for each truncated variant (Fig. 9, 11 and 13). These predictions provide an initial indication of whether key structural features are retained following truncation, which is important given the strong relationship between aptamer conformation and binding function. However, since even subtle structural changes can significantly alter binding behaviour, it was considered vital to evaluate truncation effects experimentally. Accordingly, each truncated aptamer was tested for target binding with RBCs, allowing for the direct determination of whether structural alterations influenced functional performance of each sequence to the target. The predicted secondary structures and Δ*G* values are shown in Fig. 9, 11 and 13, supporting the interpretation of the experimental findings.

For each of the three aptamers being characterised within this work, both length truncations and loop stem truncation have been explored. The first truncation method, length truncations, involved the removal of the 5′ and 3′ primer flanking regions sequentially and then simultaneously. Literature remains unclear on whether the primer flanking regions of an aptamer should be considered within the final design, so by truncating them out of the sequence, it could be determined if the removal of these regions is recommended for target binding enhancement. The second mechanism of truncation, stem loop removal, involved the removal of the hairpin loop structures within the aptamers sequence sequentially. In total, 16 truncations were tested: 5 for N1, 5 for N4 and 6 for BB1 (Table S1†). To screen these truncations for their binding affinity, ELONA was carried out as previously outlined, using isolated human RBCs as the target. This permitted for the estimation, and comparison, of *K*_D_ values for each truncation to their respective seed sequence. Within the following sections, all of the truncated sequences reported within Table 7–9 are those with highest affinity for red bloods, and thus the most suitable for further use.

#### 3.3.1. N1 aptamer truncations

The secondary structures of the five truncations experimentally trialled for N1 have been predicted using Mfold (Fig. 9)^22^ using identical temperature settings and ionic conditions used for the original aptamer sequences. All five truncations display a secondary structure with the Gibbs free energy shown in Fig. 9 for each predicted structure.

**Fig. 10:**
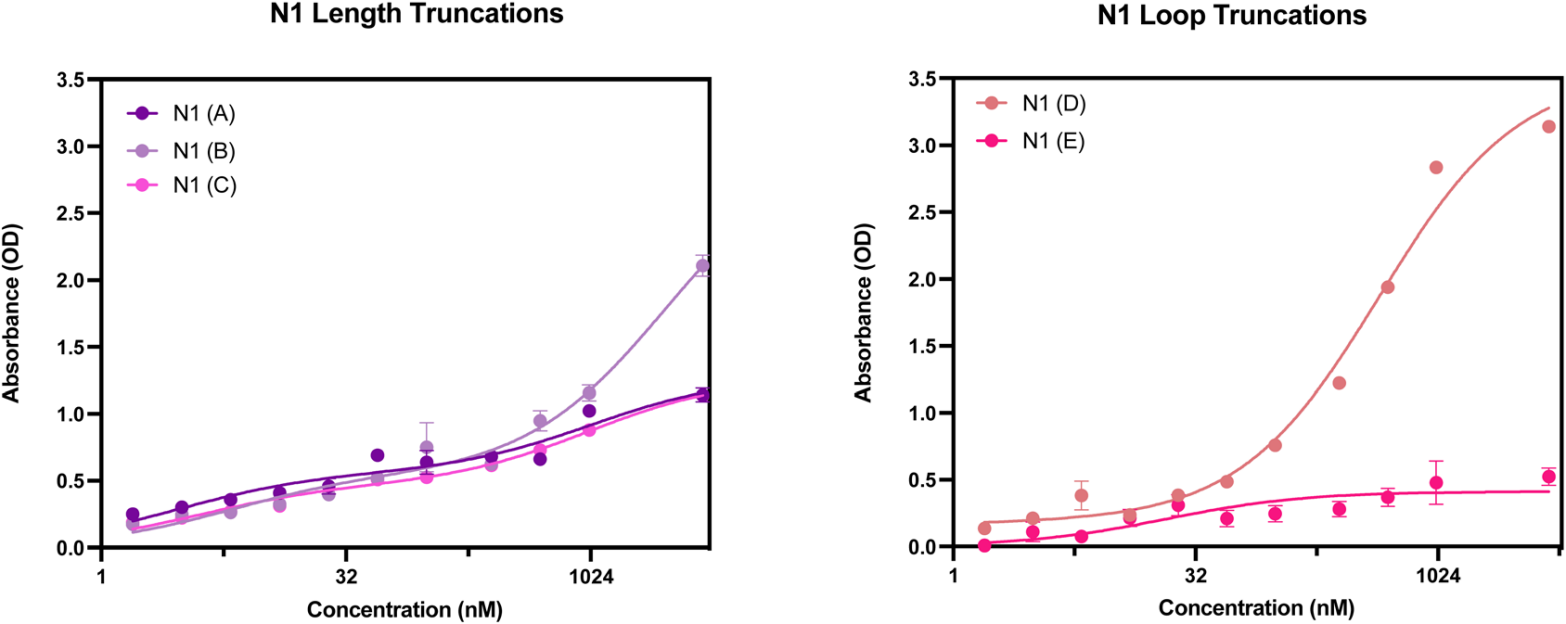
ELONA absorbance responses of interactions between a varying concentration range N1 aptamer truncations with the following sequence modifications: LEFT – (A) 5′ primer removed, (B) 3′ primer removed, (C) 5′ and 3′ primer removed, and RIGHT – (D) 5′ loop removed and (E) 3′ loop removed, and red blood cells from 3 pooled donors. Error bars = s.d.; *n* = 4 (independent experiments).

In order to screen all N1 truncations for their dissociation constant, ELONA was conducted. Fig. 10 shows the dose-dependent absorbance curves obtained from each of the five truncations when incubated with human red blood cells. In comparison with the seed N1 sequence, the three length truncations (N1(A), N1(B) and N1(C)), display a lower dose-dependent absorbance compared with the original 76 nucleotide sequence. Therefore, this is indicative of reduced binding interactions of the truncated aptamers, rendering them less suitable for use than the original sequence. Interestingly, when the 5′ loop of N1 is truncated from the sequence (N1(D)), the resulting aptamer is still able to bind to red blood cells, with an estimated *K*_D_ value that is comparable with the original seed sequence (Table 7). Whilst the dissociation constants are comparable, a shorter aptamer sequence length can be beneficial when it comes to an end application, as a shorter sequence can exhibit greater stability as well as greater sensitivity. In contrast, when the 3′ loop is removed from the seed sequence, the resulting aptamer N1(E) displays very limited binding to red blood cells (Fig. 10). This therefore indicates that the 3′ stem loop is either vital within target binding, and may form part of the binding site of N1, or may be vital for tertiary structure stability during binding.

**Table 7:** Dissociation constants for the N1 seed aptamer sequence and the promising identified truncation N1(D)

Aptamer	*K*_D_ Hi (nM)	*K*_D_ Lo (μM)	*R* ^2^
N1	1.388 ± 0.600	0.437 ± 0.037	0.998
N1(D)	0.110 ± 1.928	0.441 ± 0.093	0.986

When comparing the five N1 truncations investigated within this study, it is evident that the only truncation that is viable for further use is N1(D). This is due to the ability to bind with a high affinity in a dose-dependent manner. With a minor enhancement of the dissociation constant and shortened sequence length, the N1(D) aptamer is a promising candidate to be used as a biorecognition element for human red blood cells.

#### 3.3.2. N4 aptamer truncations

**Fig. 11:**
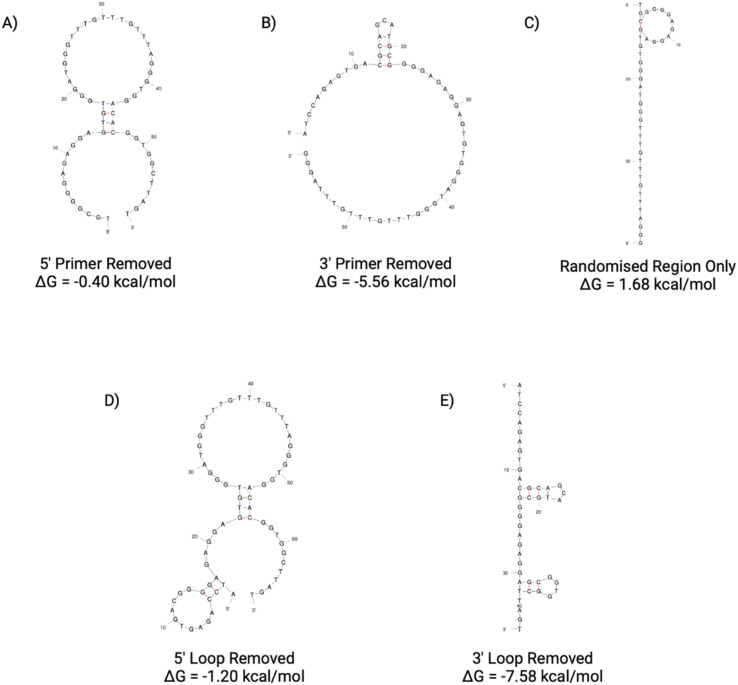
N4 aptamer truncations with the following sequence modifications: (A) 5′ primer removed, (B) 3′ primer removed, (C) 5′ and 3′ primer removed, (D) 5′ loop removed and (E) 3′ loop removed. Structures generated from Mfold software.^22^

The secondary structures of the five truncations experimentally trialled for N4 have been predicted using Mfold (Fig. 11).^22^ All five truncations display a secondary structure with the Gibbs free energy shown in Fig. 11 for each predicted structure.

**Fig. 12:**
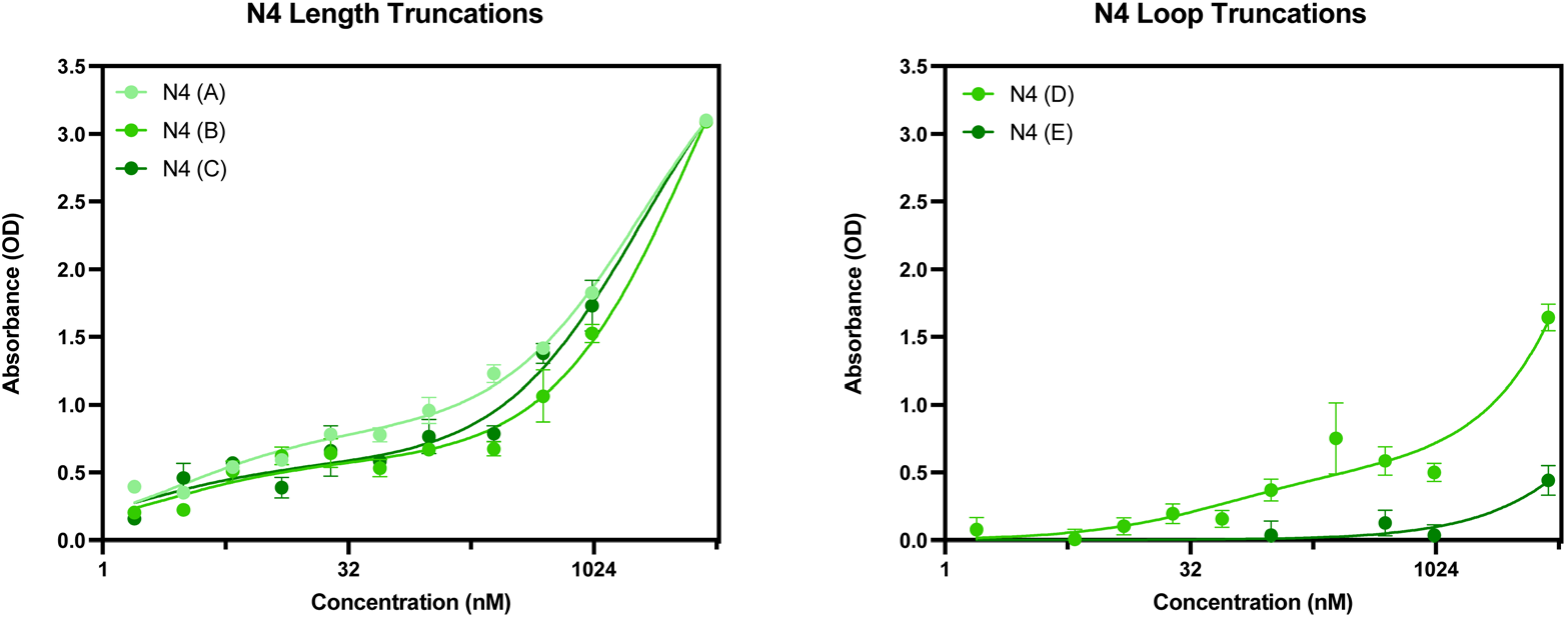
ELONA absorbance responses of interactions between a varying concentration range N4 aptamer truncations with the following sequence modifications: LEFT – (A) 5′ primer removed, (B) 3′ primer removed, (C) 5′ and 3′ primer removed, and RIGHT – (D) 5′ loop removed and (E) 3′ loop removed, and red blood cells from 3 pooled donors. Error bars = s.d.; *n* = 4 (independent experiments).

When considering the length truncations of the N4 aptamer, Fig. 12 shows that there is limited effect on the binding capabilities of the N4 truncations with human red blood cells. All three truncations (N4(A), N4(B) and N4(C)) still retain full binding capabilities with dissociation constants comparable to the seed sequence (Table 8). Whilst N4(B) displays similar *K*_D_ values as N4, it should be noted that N4(A) demonstrated a 2-fold reduction in *K*_D_ Lo and N4(C) demonstrated a 2-fold reduction in both *K*_D_ Hi and Lo constants. As these three truncations are shorter in length (58, 58 or 40 nucleotides in length), they may be more suitable than the 76 nucleotide seed sequence as it could reduce any possible non-specific binding that can be exhibited with a longer sequence. However, as shown in Fig. 12, when both the 5′ or 3′ loop are removed from the sequence, the resulting aptamer displays a significantly reduced level of binding. For these truncations, a reliable dissociation constant was unable to be estimated. Therefore, N4(D) and N4(E) are not suitable candidates for further use as an aptamer for red blood cells.

**Table 8:** Dissociation constants for the N4 seed aptamer sequence and the promising identified truncations N4(A), N4(B) and N4(C)

Aptamer	*K*_D_ Hi (nM)	*K*_D_ Lo (μM)	*R* ^2^
N4	2.958 ± 1.107	4.235 ± 1.452	0.992
N4(A)	2.974 ± 0.841	2.024 ± 0.374	0.995
N4(B)	2.302 ± 1.298	4.242 ± 1.507	0.990
N4(C)	1.637 ± 1.201	1.996 ± 0.532	0.987

Therefore, further investigation into alternative truncations for the N4 aptamer would need to be considered prior to the selection of a sequence for use in a clinical or forensic setting if sequence maturation was required. Whilst it has been shown that a large reduction in aptamer length from 76 nucleotides to 40 nucleotides (N1(C)), it remains unclear whether this binding is specific or not, as reducing aptamer length significantly can increase non-specific binding. However, it can be inferred that the 5′ and 3′ loop truncations result in non-suitable candidates due to their reduction in binding activity.

#### 3.3.3. BB1 aptamer truncations

**Fig. 13:**
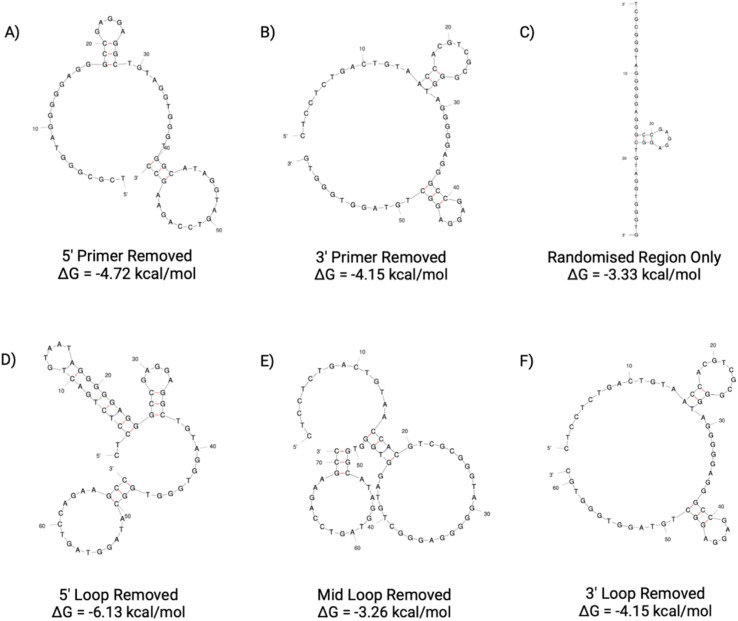
BB1 aptamer truncations with the following sequence modifications: (A) 5′ primer removed, (B) 3′ primer removed, (C) 5′ and 3′ primer removed, (D) 5′ loop removed, (E) Mid loop removed and (F) 3′ loop removed. Structures generated from Mfold software.^22^

The secondary structures of the six truncations experimentally trialled for BB1 have been predicted using Mfold (Fig. 13).^22^ All six truncations display a secondary structure with the Gibbs free energy shown in Fig. 13 for each predicted structure.

**Fig. 14:**
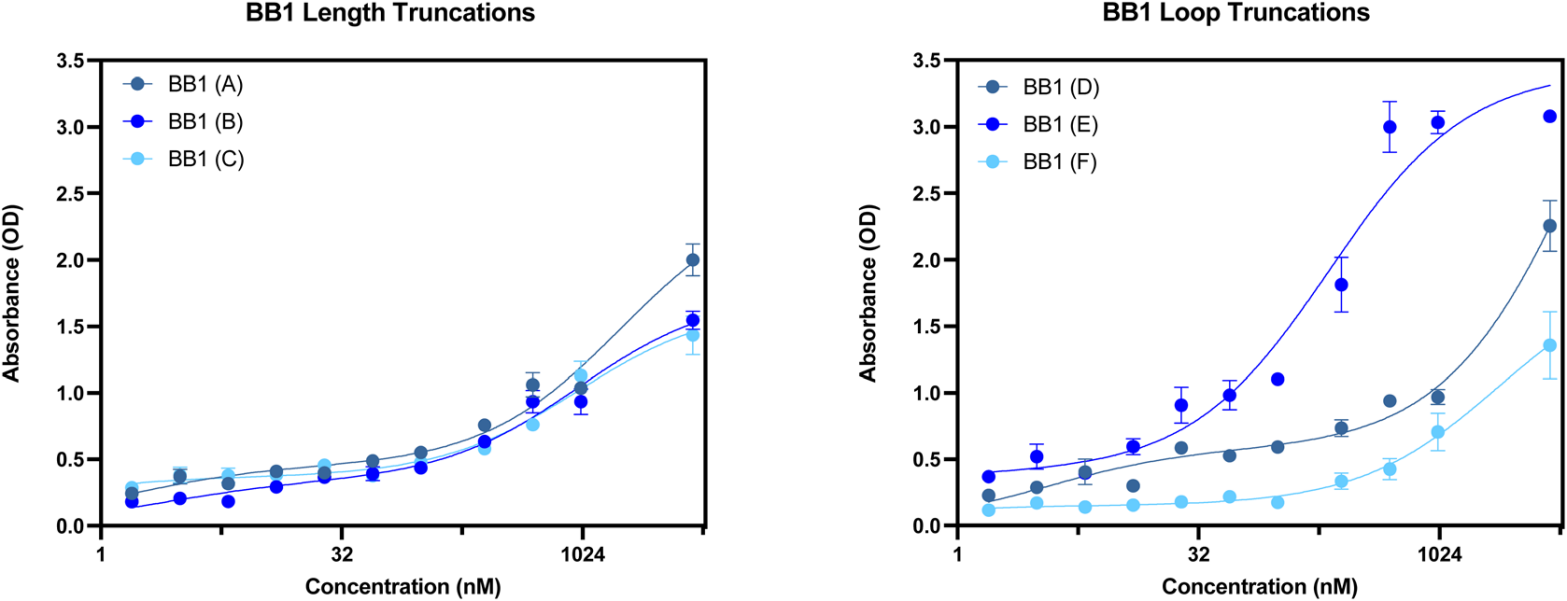
ELONA absorbance responses of interactions between a varying concentration range BB1 aptamer truncations with the following sequence modifications: LEFT – (A) 5′ primer removed, (B) 3′ primer removed, (C) 5′ and 3′ primer removed, and RIGHT – (D) 5′ loop removed, (E) Mid loop removed and (F) 3′ loop removed, and red blood cells from 3 pooled donors. Error bars = s.d.; *n* = 4 (independent experiments).

With the original BB1 sequence being 80 nucleotides in length, a truncated aptamer derivative would be beneficial in order to shorten the length and provide a more tightly folded sequence. Of the initial three length truncations trialled (BB1(A), BB1(B) and BB1(C)), a reduction in binding can be observed for each sequence when incubated with red blood cells (Fig. 14). Therefore, removing either, or both, of the primer binding regions inhibits binding to the target binding site. Similarly to the N1 aptamer loop truncation N1(D), when the middle loop of the BB1 aptamer is truncated from the sequence, there is no significant reduction in the binding affinity of the aptamer (Table 9), therefore indicating that this central loop structure on the seed sequence is not vital to structure stability or the target binding event. Reducing the sequence length from 80 to 71 nucleotides may pose additional benefits such as an increased target binding stability or specificity. Whilst binding can still be observed in a dose-dependent manner for BB1(D) and BB1(F) with the 5′ and 3′ loop structures removed, respectively, the binding is hindered when compared with the original 80 nucleotide seed sequence, so removal of these loops does not result in an enhancement of aptamer function.

**Table 9:** Dissociation constants for the BB1 seed aptamer sequence and the promising identified truncation BB1(E)

Aptamer	*K*_D_ Hi (nM)	*K*_D_ Lo (μM)	*R* ^2^
BB1	0.450 ± 0.500	0.127 ± 0.020	0.996
BB1(E)	0.094 ± 1.503	0.209 ± 0.078	0.967

When comparing the six BB1 truncations investigated within this study, it has been demonstrated that the most promising truncation is the BB1(E) aptamer. Despite the A-D and F aptamer truncations showing a level of binding to red blood cells independently, when comparing their performance to the seed BB1 sequence it is evident that binding is not enhanced. Therefore, it can be inferred that on the BB1 aptamer the 5′ and 3′ primer binding regions as well as the 5′ and 3′ loop structures are all involved in structure stability or target binding in some capacity.

## 4 Conclusion

Within this study, an extensive characterisation of three reported aptamers has been conducted for their ability to bind to human red blood cells. Previously existing gaps in the literature have been identified and features such as dissociation constants and 3-dimensional tertiary structure predictions have been estimated and presented in this work. The N1 and N4 aptamers were initially selected against whole human red blood cells, *via* a modified Cell-SELEX methodology.^18^ Therefore, their exact binding site was unreported. As outlined within this work, efforts have been made towards the resolution of possible binding sites utilising docking simulation methodology. The BB1 aptamer was initially selected again the glycophorin A protein, through a partially-robotic SELEX selection.^19^ Whilst this aptamer's binding capability to red blood cells was preliminarily demonstrated, the dissociation constant remained unreported for both the intended protein target and whole cell. By utilising HDOCK software, aptamers were screened for their docking potential to a range of red blood cell surface proteins, including glycophorin A. Results showed that the N1 and N4 aptamers shared similar docking predictions to glycophorin A, glycophorin B and glucose transporter band 4.5, with docking to be less likely for anion transporter band 3 and glycophorin C. The BB1 aptamer demonstrated a likely binding to glycophorin A, as expected, and in addition suggested that a docking event to glucose transporter band 4.5 or glycophorin B could be possible. By utilising ELONA with whole RBCs as the target, the dissociation constants of these aptamers have been calculated within the nanomolar to low micromolar range (Table 6), making them viable candidates for use within analytical assays.^9^ In addition to using RBCs as the target, both glycophorin A and glucose transporter band 4.5 were also subjected to ELONA. From the surface proteins used within HDOCK simulations, these two proteins were selected for experimental trials as they were deemed to be the most probable docking ligands for all three aptamers. Dissociation constants calculated from ELONA showed that all three aptamers were able to bind within the nanomolar to low micromolar range (Table 6). For all aptamers (N1, N4, BB1 and RDM), MST was then used to confirm the dose-dependent binding events observed during ELONA. Indeed, this confirmed the binding capabilities of N1, N4 and BB1 to human red blood cells, further demonstrating BB1's superior binding affinity to red blood cells. Therefore, this work highlights the first full characterisation of these aptamers using both *in silico* and *in vitro* methodology to identify possible ligand binding sites and subsequent calculations of dissociation constants.

Whilst these aptamers display a sufficient level of binding for use as a biorecognition element within analytical assays, efforts were made to enhance their affinity for red blood cells through affinity maturation. It was determined that of the sixteen truncations experimentally trialled for N1, N4 and BB1, only five truncations resulted in optimised sequences in terms of their dissociation constants and length. These were N1(D), N4(A), N4(B), N4(C) and BB1(E). Depending on the final use of these aptamers, it is crucial to understand if an affinity enhancement or length reduction would be of priority, as these five truncations are a combination of length reduction sequences and aptamers with an improved dissociation constant. For example, within a biosensor where aptamers may be immobilised onto a surface prior to target detection, a length truncation may be preferential to allow for a greater surface coverage, resulting in enhanced sensitivity of the assay. Alternatively, in an assay where the sample matrix may be complex, an aptamer with an improved *K*_D_ value would be preferential. Prior to use of such aptamers, further validation must be conducted specific to the end use. The research conducted within this study has been limited to the identification of potential binding sites and has not explored the possibility of cross-reactivity or non-specific binding of the aptamers to similar cell or protein targets. Therefore, when considering applications within the forensic field, it would be imperative to assess any cross-reactivity with other biological fluids or contaminants that may be encountered within a forensic setting. Similarly, within medical applications such as drug delivery or cell detection, ensuring limited non-specific binding to other cell types is vital to ensure only specific and high affinity binding is occurring.

It is hoped that the characterisation and subsequent affinity maturation of the aptamer sequences reported within this work will allow for their use within future analytical assays and open further discussions as to the use of aptamers as viable alternatives to antibodies. Within a forensic setting, it is thought that N1, N4, or BB1 could be employed as sensitive and specific biorecognition tools for the identification of human blood. The use of such aptamers could provide an improvement over traditional serological or immunological methods, resulting in higher specificity and sensitivity of detection. However, for use in forensic applications, further testing and validation would be required to assess key performance parameters, including the limit of detection (LOD), specificity in the presence of environmental contaminants, and potential cross-reactivity with non-human blood samples. Establishing the robustness of these aptamers under varied field conditions would be essential before integration into forensic workflow. Beyond analytical and forensic contexts, these aptamers also show promise in clinical and therapeutic applications. For example, their ability to bind RBCs specifically opens opportunities for their use in targeted drug delivery systems. By conjugating these aptamers to therapeutic agents, it may be possible to achieve RBC-targeted delivery of drugs, improving pharmacokinetics and reducing off-target effects. Additionally, the aptamers could be used as diagnostic tools for tagging or monitoring RBCs in various haematological conditions, including sickle cell disease, thalassaemia, or haemolytic anaemia. Their small size, low immunogenicity, and ease of chemical modification give them a practical advantage over antibodies in such applications. Furthermore, aptamers can be readily adapted for a range of purposes, such as the attachment of signalling molecules, therapeutic agents, or other functional components, which broadens their potential use in both therapeutic and diagnostic settings. While this study focuses on initial characterisation and optimisation, the broader potential of N1, N4, and BB1 should not be limited to the examples highlighted here. These sequences may be suitable for a wide variety of innovative applications involving human blood.

Where previously there has been limited aptamer reporting for red blood cell binding candidates, this work aimed to provide supporting evidence for the use of the three identified candidates, whilst demonstrating how *in silico* methodologies in tandem with experimental research can enhance one another to give a more detailed understanding of the structure and binding mechanisms of aptamer sequences.

## Ethical statement

All bodily fluid sample collection and use within this study was conducted in accordance with ethical clearance granted by the King's College London Biomedical Sciences, Dentistry, Medicine and Natural & Mathematical Sciences Research Ethics Subcommittee (Reference HR-17/18-5057). All research was conducted in accordance with the Human Tissue Act 2004.

## Consent to participate

Informed consent was obtained from all donors involved in the study.

## Author contributions

Conceptualization, Hayley Costanzo, Nunzianda Frascione, James Gooch; methodology, Hayley Costanzo, James Gooch; formal analysis, Hayley Costanzo; investigation, Hayley Costanzo; data curation, Hayley Costanzo; writing – original draft preparation, Hayley Costanzo; writing – review & editing, Hayley Costanzo, Nunzianda Frascione & James Gooch; supervision, Nunzianda Frascione & James Gooch; funding acquisition, Nunzianda Frascione.

## Conflicts of interest

The authors declare no conflict of interest.

## Supplementary Material

RA-015-D5RA00645G-s001

## Data Availability

All data used and analysed within this work has been reported. Further data is available within the ESI.†
