# Supplementary material for: In silico and in vitro characterisation and affinity maturation of human red blood cell binding aptamers
Source: RSC Adv. 2025 Jul 1;15(28):22505–23. doi: 10.1039/d5ra00645g (PMC12211734; doi:10.1039/d5ra00645g)
Supplement: RA-015-D5RA00645G-s001 [file RA-015-D5RA00645G-s001.pdf]

This journal is © The Royal Society of Chemistry 2025

Supplementary Information:

Table S1. Aptamer sequences of truncations of N1, N4 and BB1. Aptamer truncations are outlined by their unique identifier, truncation mechanism, length in bases and sequence.

Aptamer Identifier	Truncation	Length (bases)	Sequence (5' - 3')
**N1 (A)**	5' Primer Removed	58	CGGGTTGGGGCTGGTTGTGTGTTGTTTTTTTGGCTGTATGTGGACACGGTGGCT TAGT
**N1 (B)**	3' Primer Removed	58	ATCCAGAGTGACGCAGCACGGGTTGGGGCTGGTTGTGTGTTGTTTTTTTGGCTG TATG
**N1 (C)**	5' & 3' Primer Removal	40	CGGGTTGGGGCTGGTTGTGTGTTGTTTTTTTGGCTGTATG
**N1 (D)**	5' Loop Removed	60	ATCCAGAGTGACGCGGTTGTGTGTTGTTTTTTTGGCTGTATGTGGACACGGTGG CTTAGT
**N1 (E)**	3' Loop Removed	49	ATCCAGAGTGACGCAGCACGGGTTGGGGCTGGTTGTGCGGTGGCTTAGT
**N4 (A)**	5' Primer Removed	58	TGCGGGGAGAGGAGTGTGGGATGGGTTTGTTTGTTTAGGGTGGACACGGTGGC TTAGT
**N4 (B)**	3' Primer Removed	58	ATCCAGAGTGACGCAGCATGCGGGGAGAGGAGTGTGGGATGGGTTTGTTTGTTT AGGG
**N4 (C)**	5' & 3' Primer Removal	40	TGCGGGGAGAGGAGTGTGGGATGGGTTTGTTTGTTTAGGG
**N4 (D)**	5' Loop Removed	67	ATCCAGAGTGACGGGGAGAGGAGTGTGGGATGGGTTTGTTTGTTTAGGGTGGA CACGGTGGCTTAGT
**N4 (E)**	3' Loop Removed	44	ATCCAGAGTGACGCAGCATGCGGGGAGAGGAGCGGTGGCTTAGT

**BB1 (A)**	5’ Primer Removed	60	TCGCGGGTAGGGGGAGGGCCGAGGAGGCTGTAGGTGGGTGGCATAGGTAGTCCAGAAGCC
**BB1 (B)**	3’ Primer Removed	60	CTCCTCTGACTGTAACCACGTCGCGGGTAGGGGGAGGGCCGAGGAGGCTGTAGGTGGGTG
**BB1 (C)**	5’ & 3’ Primer Removal	40	TCGCGGGTAGGGGGAGGGCCGAGGAGGCTGTAGGTGGGTG
**BB1 (D)**	5’ Loop Removed	68	CTCCTCTGACTGTAATAGGGGGAGGGCCGAGGAGGCTGTAGGTGGGTGGCATAGGTAGTCCAGAAGCC
**BB1 (E)**	Mid Loop Removed	71	CTCCTCTGACTGTAACCACGTCGCGGGTAGGGGGAGGGCTGTAGGTGGGTGGCATAGGTAGTCCAGAAGCC
**BB1 (F)**	3’ Loop Removed	61	CTCCTCTGACTGTAACCACGTCGCGGGTAGGGGGAGGGCCGAGGAGGCTGTAGGTGGGTGC

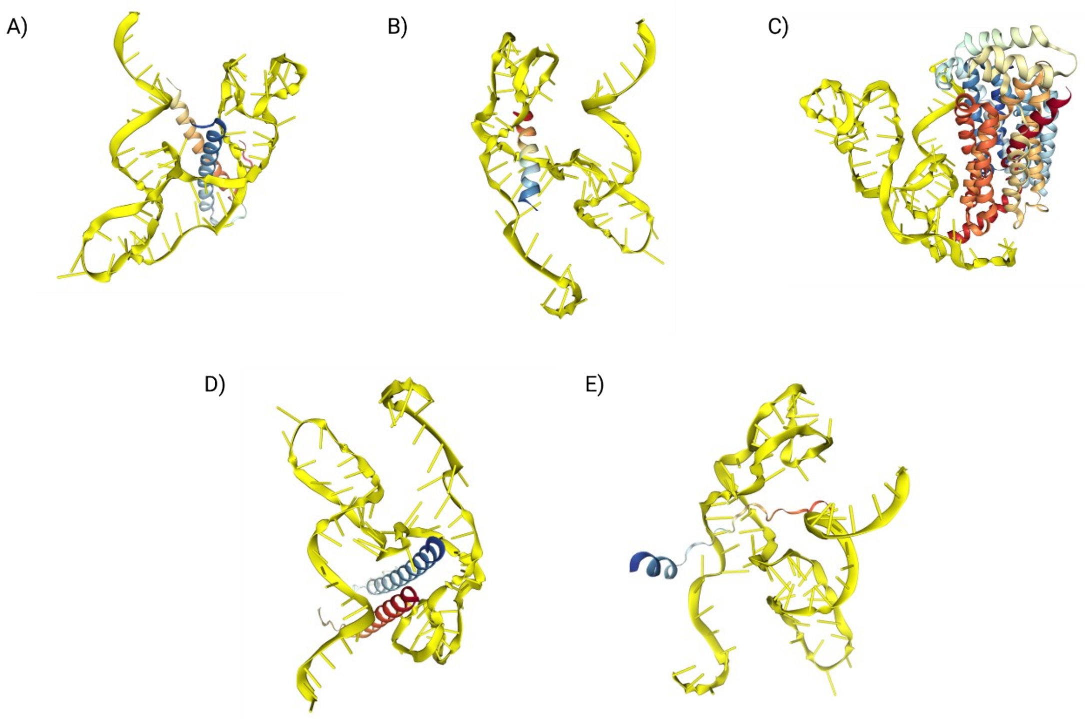

Figure S1. HDOCK docking models for N1 with RBC surface proteins. A) N1 + Glycophorin A, B) N1 + Band 3 Anion Transporter, C) N1 + Band 4.5 Glucose Transporter, D) Glycophorin B and E) Glycophorin C.

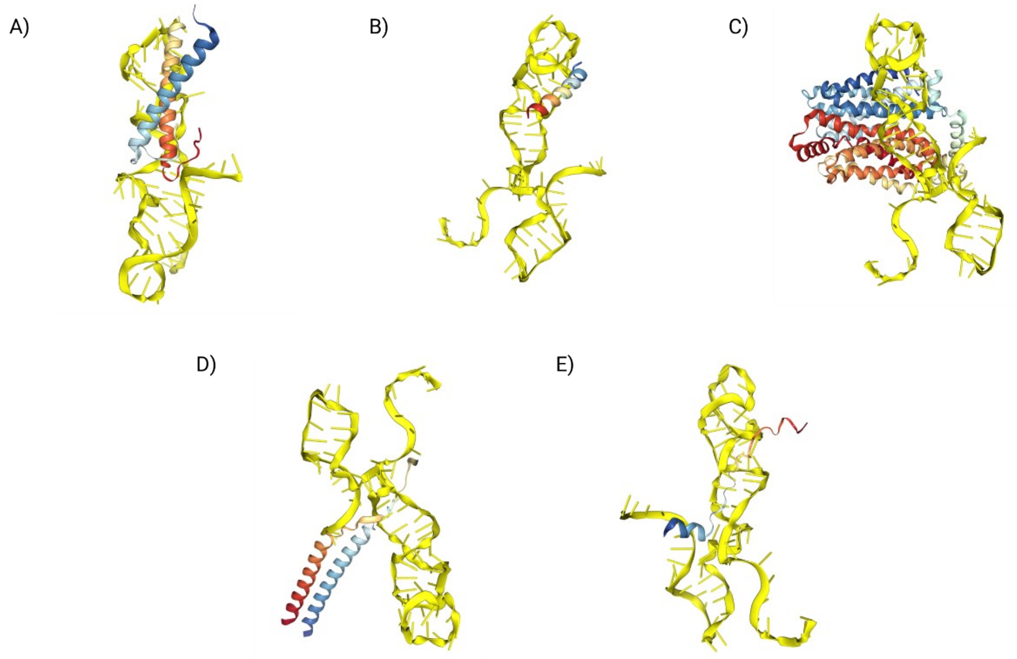

Figure S2. HDOCK docking models for N4 with RBC surface proteins. A) N4 + Glycophorin A, B) N4 + Band 3 Anion Transporter, C) N4 + Band 4.5 Glucose Transporter, D) Glycophorin B and E) Glycophorin C.

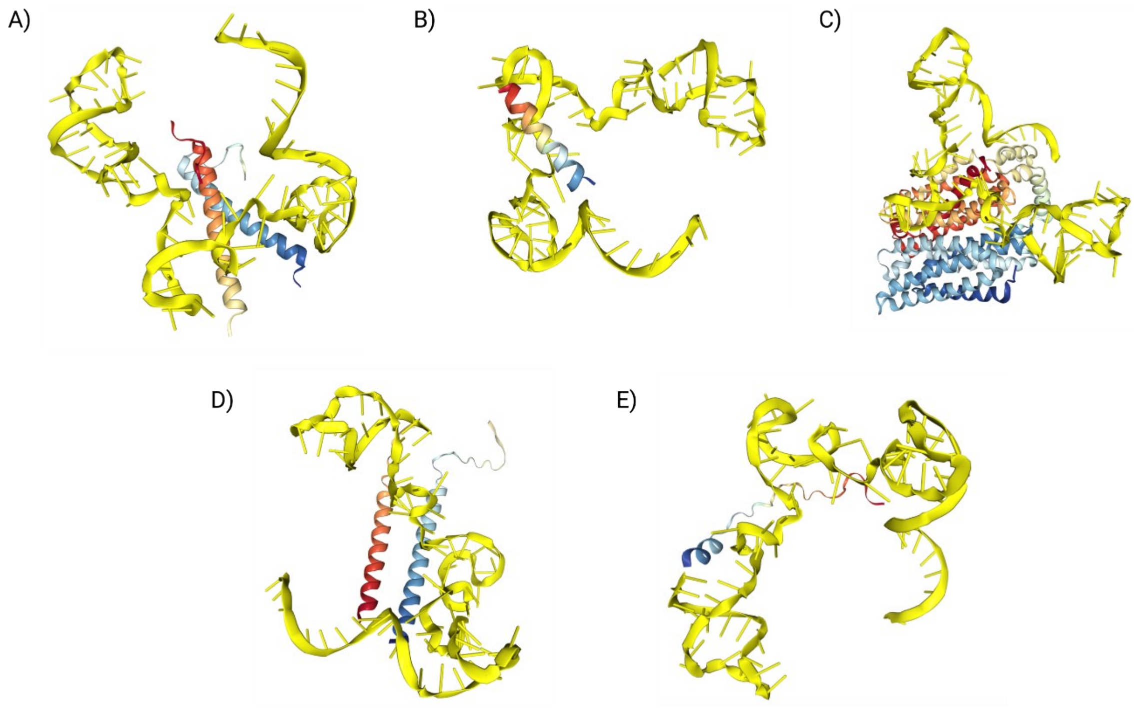

Figure S3. HDOCK docking models for BB1 with RBC surface proteins. A) BB1 + Glycophorin A, B) BB1 + Band 3 Anion Transporter, C) BB1 + Band 4.5 Glucose Transporter, D) Glycophorin B and E) Glycophorin C.

Table S2. ELONA absorbance responses of interactions between a varying concentration range of aptamers N1, N4, BB1 and RDM and red blood cells. The mean and SEM are given. n = 4 (independent experiments).

	N1		N4		BB1		RDM	
**Aptamer Concentration (nM)**	**Mean**	**SEM**	**Mean**	**SEM**	**Mean**	**SEM**	**Mean**	**SEM**
0	0.000	0.009	0.000	0.010	0.000	0.021	0.000	0.006
1.5625	0.311	0.044	0.293	0.010	0.553	0.040	0.010	0.008
3.125	0.401	0.009	0.299	0.023	0.582	0.031	0.004	0.004
6.25	0.439	0.011	0.450	0.075	0.744	0.051	0.009	0.004
12.5	0.512	0.015	0.427	0.053	0.901	0.015	0.012	0.004
25	0.61	0.045	0.492	0.032	1.149	0.060	0.016	0.005

	8							
50	0.850	0.025	0.584	0.056	1.261	0.076	0.021	0.005
100	1.045	0.048	0.770	0.036	1.757	0.151	0.033	0.007
250	1.533	0.022	0.873	0.087	2.385	0.057	0.042	0.005
500	2.012	0.069	1.035	0.050	2.845	0.045	0.07	0.006
1000	2.351	0.085	1.232	0.037	3.032	0.019	0.099	0.006
5000	3.084	0.014	2.559	0.067	3.092	0.038	0.285	0.011

Table S3. ELONA absorbance responses of interactions between a varying concentration range of aptamers N1, N4 and BB1 and Glycophorin A. The mean and SEM are given. n = 3 (independent experiments).

	N1		N4		BB1	
**Aptamer Concentrati**	**Mea**	**SEM**	**Mea**	**SEM**	**Mean**	**SEM**

on (nM)	n		n			
0	0.000	0.161	0.000	0.091	0.000	0.082
1.5625	-0.132	0.169	0.020	0.092	0.120	0.104
3.125	0.133	0.191	-0.095	0.260	0.355	0.209
6.25	0.198	0.199	0.202	0.107	0.505	0.221
12.5	0.127	0.168	0.156	0.175	0.856	0.246
25	0.444	0.278	0.783	0.065	1.224	0.165
50	0.602	0.224	0.814	0.350	1.306	0.423
100	0.55	0.207	0.48	0.361	1.324	0.065

	2		7			
250	0.689	0.191	0.569	0.175	1.424	0.305
500	1.518	0.440	1.078	0.375	1.535	0.310
1000	2.029	0.160	0.942	0.272	2.360	0.126
5000	2.325	0.199	2.058	0.212	2.656	0.174

Table S4. ELONA absorbance responses of interactions between a varying concentration range of aptamers N1, N4 and BB1 and Glucose Transporter Band 4.5. The mean and SEM are given. n = 3 (independent experiments).

	N1		N4		BB1	
**Aptamer Concentration (nM)**	**Mean**	**SEM**	**Mean**	**SEM**	**Mean**	**SEM**
0	0.000	0.029	0.000	0.032	0.000	0.004
1.5625	0.275	0.098	0.223	0.063	0.265	0.105
3.125	0.309	0.091	0.482	0.082	0.304	0.067

6.25	0.528	0.102	0.683	0.141	0.443	0.042
12.5	0.624	0.025	0.778	0.041	0.328	0.032
25	0.950	0.286	1.025	0.051	0.698	0.085
50	1.057	0.119	1.392	0.141	1.198	0.138
100	1.219	0.212	1.592	0.101	1.727	0.124
250	2.090	0.184	1.757	0.148	1.981	0.169
500	2.120	0.305	2.114	0.292	1.949	0.404
1000	2.427	0.117	2.602	0.138	2.255	0.295
5000	2.662	0.178	2.887	0.109	2.094	0.040

Table S5. MST responses of interactions between aptamers N1, N4, BB1 and RDM with serially diluted red blood cell suspensions. The mean and SEM are given. n = 3 (independent experiments).

	N1	N4	BB1	RDM

Red Blood Cells (per mL)	Mean	SEM	Mean	SEM	Mean	SEM	Mean	SEM
**5.00×10^8**	-49.116	3.315	-38.106	2.607	-58.188	1.175	-11.254	1.200
**2.50×10^8**	-44.436	3.718	-18.245	1.051	-48.243	4.884	-4.434	0.167
**1.25×10^8**	-16.988	2.590	-6.545	1.380	-18.873	1.468	-7.968	7.146
**6.25×10^7**	-7.630	3.437	-4.020	0.908	-10.140	2.917	-0.255	0.913
**3.13×10^7**	-5.760	2.782	-1.256	1.819	-3.166	4.634	-0.257	0.786
**1.56×10^7**	-2.354	0.402	-2.583	0.410	-4.596	0.334	0.051	1.864
**7.81×10^6**	-2.615	3.467	-2.569	1.521	-3.331	0.399	-1.726	1.776
**3.91×10^6**	-4.825	1.378	-1.377	0.719	-4.172	1.775	0.045	2.273

Table S6. ELONA absorbance responses of interactions between a varying concentration range of the N1 aptamer truncations and red blood cells The mean and SEM are given. n = 4 (independent experiments).

	N1 (A)		N1 (B)		N1 (C)		N1 (D)		N1 (E)	
**Aptamer Concentration (nM)**	**Mean**	**SEM**	**Mean**	**SEM**	**Mean**	**SEM**	**Mean**	**SEM**	**Mean**	**SEM**
0	0.000	0.005	0.000	0.011	0.000	0.004	0.000	0.021	0.000	0.023
1.5625	0.250	0.005	0.173	0.011	0.186	0.009	0.136	0.017	0.010	0.029
3.125	0.301	0.025	0.246	0.039	0.221	0.015	0.210	0.019	0.109	0.074
6.25	0.357	0.021	0.261	0.022	0.270	0.015	0.383	0.107	0.076	0.039
12.5	0.407	0.016	0.323	0.030	0.310	0.018	0.235	0.032	0.214	0.064
25	0.457	0.057	0.395	0.037	0.458	0.014	0.384	0.036	0.310	0.080
50	0.690	0.041	0.519	0.043	0.509	0.031	0.486	0.018	0.209	0.061
100	0.638	0.087	0.751	0.185	0.525	0.055	0.759	0.045	0.246	0.062
250	0.681	0.029	0.625	0.017	0.614	0.088	1.223	0.043	0.279	0.057
500	0.661	0.041	0.948	0.075	0.728	0.088	1.940	0.044	0.369	0.067

1000	1.022	0.046	1.156	0.060	0.880	0.107	2.836	0.050	0.479	0.163
5000	1.139	0.052	2.108	0.080	1.138	0.024	3.141	0.020	0.523	0.066

Table S7. ELONA absorbance responses of interactions between a varying concentration range of the N4 aptamer truncations and red blood cells The mean and SEM are given. n = 4 (independent experiments).

	N4 (A)		N4 (B)		N4 (C)		N4 (D)		N4 (E)	
**Aptamer Concentration (nM)**	**Mean**	**SEM**	**Mean**	**SEM**	**Mean**	**SEM**	**Mean**	**SEM**	**Mean**	**SEM**
0	0.000	0.031	0.000	0.043	0.000	0.020	0.000	0.088	0.000	0.101
1.5625	0.395	0.049	0.203	0.032	0.157	0.031	0.077	0.088	-0.080	0.084
3.125	0.351	0.030	0.223	0.030	0.459	0.109	-0.028	0.078	-0.161	0.075
6.25	0.540	0.039	0.509	0.074	0.569	0.032	0.008	0.070	-0.092	0.072
12.5	0.592	0.030	0.623	0.066	0.387	0.076	0.102	0.063	-0.069	0.074
25	0.782	0.031	0.643	0.107	0.659	0.187	0.193	0.074	-0.039	0.077

50	0.780	0.053	0.532	0.063	0.583	0.049	0.155	0.064	-0.045	0.077
100	0.959	0.097	0.669	0.044	0.766	0.127	0.369	0.080	0.034	0.107
250	1.229	0.065	0.674	0.052	0.788	0.060	0.752	0.262	-0.012	0.097
500	1.417	0.041	1.064	0.192	1.379	0.075	0.585	0.106	0.125	0.094
1000	1.828	0.048	1.527	0.067	1.731	0.189	0.500	0.067	0.035	0.077
5000	3.102	0.029	3.092	0.039	3.098	0.028	1.644	0.100	0.442	0.110

Table S8. ELONA absorbance responses of interactions between a varying concentration range of the BB1 aptamer truncations and red blood cells The mean and SEM are given. n = 4 (independent experiments).

	BB1 (A)		BB1 (B)		BB1 (C)		BB1 (D)		BB1 (E)		BB1 (F)	
**Aptamer Concentration (nM)**	**Mean**	**SEM**	**Mean**	**SEM**	**Mean**	**SEM**	**Mean**	**SEM**	**Mean**	**SEM**	**Mean**	**SEM**
0	0.000	0.008	0.000	0.021	0.000	0.016	0.000	0.006	0.000	0.012	0.000	0.019

1.5625	0.243	0.007	0.181	0.022	0.286	0.041	0.229	0.011	0.369	0.034	0.116	0.028
3.125	0.370	0.055	0.207	0.015	0.382	0.058	0.289	0.021	0.520	0.094	0.170	0.045
6.25	0.317	0.016	0.182	0.019	0.378	0.056	0.406	0.096	0.396	0.046	0.141	0.028
12.5	0.409	0.034	0.291	0.030	0.382	0.016	0.299	0.047	0.596	0.061	0.154	0.037
25	0.399	0.025	0.366	0.035	0.456	0.042	0.585	0.026	0.908	0.134	0.179	0.049
50	0.488	0.024	0.392	0.053	0.378	0.019	0.527	0.012	0.984	0.110	0.217	0.028
100	0.553	0.015	0.436	0.018	0.477	0.031	0.592	0.046	1.103	0.042	0.174	0.017
250	0.759	0.047	0.635	0.027	0.581	0.029	0.736	0.064	1.814	0.207	0.335	0.061
500	1.060	0.091	0.935	0.085	0.761	0.028	0.941	0.031	3.000	0.190	0.426	0.079

1000	1.037	0.046	0.936	0.096	1.130	0.107	0.969	0.057	3.034	0.085	0.706	0.141
5000	2.002	0.121	1.546	0.068	1.434	0.145	2.255	0.188	3.081	0.017	1.358	0.253
